# Intron location and sequence modulate gene expression in *Yarrowia lipolytica*

**DOI:** 10.1093/nar/gkag650

**Published:** 2026-06-27

**Authors:** Qi Qi, Pedro Tomaz da Silva, Vasileios Vangalis, Seppe Dockx, Jan Steensels, Karin Voordeckers, Julien Gagneur, Kevin J Verstrepen

**Affiliations:** Centre of Microbial and Plant Genetics (CMPG), Department of Microbial and Molecular Systems (M^2^S), KU Leuven, Leuven 3000, Belgium; Lab for Systems Biology, VIB Center for Microbiology, VIB, Leuven 3001, Belgium; School of Computation, Information and Technology, Technical University of Munich, Munich 80333, Germany; Munich Center for Machine Learning, Munich 80333, Germany; Centre of Microbial and Plant Genetics (CMPG), Department of Microbial and Molecular Systems (M^2^S), KU Leuven, Leuven 3000, Belgium; Lab for Systems Biology, VIB Center for Microbiology, VIB, Leuven 3001, Belgium; Centre of Microbial and Plant Genetics (CMPG), Department of Microbial and Molecular Systems (M^2^S), KU Leuven, Leuven 3000, Belgium; Lab for Systems Biology, VIB Center for Microbiology, VIB, Leuven 3001, Belgium; Centre of Microbial and Plant Genetics (CMPG), Department of Microbial and Molecular Systems (M^2^S), KU Leuven, Leuven 3000, Belgium; Lab for Systems Biology, VIB Center for Microbiology, VIB, Leuven 3001, Belgium; Centre of Microbial and Plant Genetics (CMPG), Department of Microbial and Molecular Systems (M^2^S), KU Leuven, Leuven 3000, Belgium; Lab for Systems Biology, VIB Center for Microbiology, VIB, Leuven 3001, Belgium; School of Computation, Information and Technology, Technical University of Munich, Munich 80333, Germany; Institute of Human Genetics, School of Medicine and Health, Technical University of Munich, Munich 81675, Germany; Computational Health Center, Helmholtz Center Munich, Neuherberg 85764, Germany; Centre of Microbial and Plant Genetics (CMPG), Department of Microbial and Molecular Systems (M^2^S), KU Leuven, Leuven 3000, Belgium; Lab for Systems Biology, VIB Center for Microbiology, VIB, Leuven 3001, Belgium

## Abstract

Introns are widespread among eukaryotic genomes. While intron-containing genes often show higher expression than genes lacking introns, the intron features influencing gene expression remain largely elusive. Here, we systematically characterize the intron landscape of *Yarrowia lipolytica*, an oleaginous yeast that is increasingly used as a microbial cell factory. Transcriptome analysis across 12 environments identified 2421 introns in 1430 genes, including 1302 newly discovered introns and 479 newly annotated intron-containing genes. We find that intron-containing genes exhibit higher and more stable expression across conditions and identify six key intron features, including the 5′ splice motif, 3′ splice motif, branch point motif, distance from branch point to 3′ splice site, GC content, and intron size, that influence splicing efficiency and gene expression. A linear regression model based on these features robustly captures the intron’s effect on gene expression, enabling us to select and test 55 different introns that modulate expression of a reporter gene by 200-fold. Moreover, we demonstrate that intron effects are robust across genomic contexts and identify a previously uncharacterized intron, I3, that strongly enhances gene expression and protein production. Together, our results provide new fundamental insights and open new avenues for using introns as regulatory elements.

## Graphical Abstract

**Graphical Abstract:**
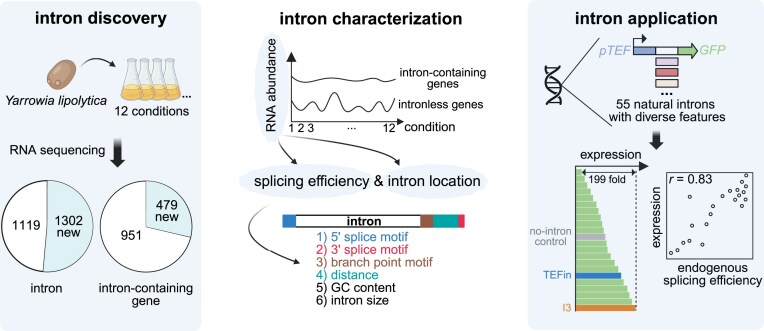

## Introduction

Introns are non-coding sequences that are found in almost all eukaryotic genomes [1]. They are typically spliced out during precursor RNA maturation, a process guided by three key intron splice signals: the 5′ splice site, branch point, and 3′ splice site [2]. While initially regarded as a mechanism to allow the production of multiple protein isoforms from one gene [3], it is now known that the presence of introns and splicing also contributes to gene expression regulation. Since introns can contain potential start or stop codons, their retention in the 5′ untranslated region (5′UTR) of genes may generate upstream open reading frames (uORFs) that suppress translation of the main open reading frame (ORF) [4, 5], while retention in the ORF could introduce premature stop codons, potentially triggering nonsense-mediated decay of the transcripts [6]. Conversely, introns and splicing can sometimes enhance gene expression and protein expression, a process called intron-mediated enhancement (IME) [7, 8]. This effect is thought to be driven by a combination of factors including intron-dependent gene looping [9], promoter-proximal splicing-dependent transcription regulation [10], exon junction complex (EJC)-facilitated nuclear export [11, 12], and EJC-associated regulation of messenger RNA (mRNA) stability and translation [13].

Despite the prevalence of introns in eukaryotic genomes, the exact intron features that determine whether and how strongly an intron influences gene and protein expression are still largely unknown. Here, we set out to investigate which intron features affect gene regulation using yeasts, single-cell eukaryotes with relatively small genomes that have proven to be useful models for general eukaryotic biology. Although yeasts are considered intron-poor organisms, accumulating evidence shows that introns play important roles in regulating gene expression, coordinating stress responses, and promoting cell survival during starvation and other stress conditions [14–18]. For example, in the model organism *Saccharomyces cerevisiae*, 240 native introns were inserted into a synthetic reporter gene, and most were found to reduce expression levels due to incomplete splicing [19]. In contrast, placing introns adjacent to the start codon of a reporter gene expanded the dynamic range of a promoter library from 2.4- to 7-fold [20], and combining the *TDH3* promoter with the *RPS25A* intron led to a 50-fold increase in gene expression [21], with the IME found to depend on conserved 5′ splice site and branch point motifs [22].

Whereas *S. cerevisiae* offers the advantage of being a tractable genetic model system for which an expansive molecular toolbox is available, it may not be the best organism to study the natural physiological role of introns because only ~5% of *S. cerevisiae* genes contain an intron [23]. In contrast, introns are more common in the oleaginous yeast *Yarrowia lipolytica*. Among hemiascomycetes, *Y. lipolytica* possesses the highest number of introns identified to date [6], and the limited data available suggest that at least some introns have an important effect on gene expression [24, 25]. The most commonly studied intron in *Y. lipolytica* is the *TEF* intron. Inserting the *TEF* intron immediately downstream of the start codon of a reporter gene under the control of the *TEF* promoter led to a 17-fold increase in gene expression when an episomal plasmid was used [26], and a 15%–85.9% increase with single-copy genomic integration across different genomic loci and growth conditions [27, 28].

Another reason to study introns in *Y. lipolytica* is that this yeast is increasingly used as a metabolic model and an industrial cell factory [29–31]. It has a strong lipogenic central metabolism and can use a wide range of substrates, including hydrophilic compounds such as glucose and glycerol, as well as hydrophobic ones such as oleic acid and alkanes [32, 33], making it an attractive chassis strain for multiple industrial biotechnology applications. Several large-scale industrial precision fermentation processes that use *Y. lipolytica* have recently been started [34]. However, despite its considerable potential as a chassis for metabolic engineering, there is currently only a very limited toolbox to tune gene expression in *Y. lipolytica* [29, 35]. In fact, the *TEF* promoter–intron combination (pTEFin) has emerged as one of the few strong promoters that are commonly used to engineer *Y. lipolytica* [36–39].

In this study, we used *Y. lipolytica* to systematically investigate intron-mediated regulation, obtaining insight into the key factors that influence splicing efficiency and gene expression. We first characterized the intron landscape of *Y. lipolytica* by analyzing transcriptomes across 12 different, biotechnologically relevant growth conditions that varied by carbon source (glucose, glycerol, or oleic acid), carbon-to-nitrogen ratio, and growth phase (exponential or early stationary). This intron annotation enabled us to investigate how intron presence, splicing efficiency, genomic location, and sequence features are associated with gene expression. By integrating intron feature analysis, predictive modeling, and reporter-based validation, we further assessed whether introns can serve as modular regulatory elements across genomic contexts. Taken together, our results provide insights into the key features affecting IME and support the application of introns as regulatory elements in synthetic biology and metabolic engineering.

## Materials and methods

### Strains, plasmids, and oligonucleotides

All *Y. lipolytica* strains used in this study were derived from type strain W29 (ATCC 20460). The starting strain Y01 (W29, *Δku70::pTDH1-hphB-tPEX20*) was constructed to enhance the efficiency of homologous recombination and subsequently used for genome editing. The full strain list can be found in Supplementary Table S1. Yeast transformation was performed using a LiAc/SS DNA/PEG method, incorporating a CRISPR/Cas9-mediated genome editing strategy, based on a direct tRNA–sgRNA fusion system [40].

All plasmids used in this study were constructed using Gibson Assembly (NEBuilder HiFi DNA Assembly Master Mix). *Escherichia coli* strain DH5α (NEB) was used for plasmid construction and transformation. The full plasmid list can be found in Supplementary Table S2.

All DNA oligonucleotides used in this study were synthesized by Integrated DNA Technologies. A full list can be found in Supplementary Table S3.

### Media and culture conditions

For yeast transformation, yeast peptone dextrose medium was prepared with 10 g/l yeast extract (Neogen, USA), 20 g/l peptone (Neogen), and 20 g/l glucose (Millipore, Germany). The selection antibiotics hygromycin B (Invitrogen, USA) and nourseothricin (Jena Bioscience, Germany) were added at 100 mg/l and 250 mg/l, respectively, if needed. For *E. coli* transformation, Luria–Bertani medium was prepared with 5 g/l yeast extract (Neogen), 10 g/l tryptone (Neogen), and 10 g/l sodium chloride (Sigma–Aldrich, Germany). The selection antibiotic carbenicillin (Duchefa Biochemie, The Netherlands) was added at 100 mg/l if needed. For solid media, bacteriological agar (VWR, USA) was added at 20 g/l.

For RNA sequencing sample collection, a defined fermentation medium was prepared using yeast nitrogen base without amino acids and ammonium sulfate (Formedium, UK) at 1.7 g/l. The carbon source was provided at an equivalent level of 1 mol carbon per liter, using either glucose (glc, Millipore) at 30 g/l, glycerol (gly, Chem-Lab, Belgium) at 30.7 g/l, or oleic acid (oa, Merck, Germany) at 15.7 g/l. For oleic acid, 0.1% Tween 80 (Sigma–Aldrich) was added as an emulsifier. The nitrogen source was supplied as ammonium sulfate (Sigma–Aldrich) at either 6.6 g/l or 0.55 g/l to achieve carbon-to-nitrogen (C/N) ratios of 10 or 120, respectively. In total, six media conditions were used, based on the carbon source and C/N ratio: glc10 (glucose as the carbon source and a C/N ratio of 10; similar for the others), glc120, gly10, gly120, oa10, and oa120. The media condition glc120 was further used for *hrGFP* and *lacZ* strain cultivation.

For RNA sequencing sample collection, fresh colonies of *Y. lipolytica* strain W29 were inoculated into 3 ml of one of the six media conditions described above, respectively, and cultured overnight at 30°C in 14-ml test tubes using a tube rotator (New Brunswick Scientific, Germany). The resulting seed cultures were then inoculated into 50 ml of the corresponding media in 250-ml Erlenmeyer flasks at an initial OD_600_ of 0.05. Cultivations were carried out at 30°C and 220 rpm. Samples were harvested after 24 and 72 h of cultivation, corresponding to exponential phase and early stationary phase.

For fluorescence intensity measurement of *hrGFP* strains, fresh colonies of the corresponding strains were inoculated into 150 µl glc120 medium in a 96-well plate (CELLSTAR 96 well plate V bottom, Greiner Bio-One, Austria) and cultured at 30°C and 900 rpm overnight to obtain seed cultures. These seed cultures were then diluted into fresh glc120 medium at a ratio of 10 µl seed culture to 140 µl medium. To minimize edge effects caused by evaporation and potential variation in oxygen availability [41], the first and last rows and columns of all 96-well plates were filled with sterile glc120 medium and used as cell-free humidifiers; only the remaining inner wells were used for cultivation of yeast strains. A plate seal (Microseal B seal optically clear, Bio-Rad, USA) was applied to prevent medium evaporation. Cultivations were carried out at 30°C and 900 rpm using a microplate rotator (Heidolph, Germany). After 16 h of cultivation, cells were harvested for *hrGFP* fluorescence intensity measurements, as described in the “Fluorescence analysis” section.

For β-galactosidase activity measurement of *lacZ* strains, fresh colonies of the corresponding strains were inoculated into 1 ml glc120 medium in a 24-well plate (Cell culture plate, 24 well, flat base, Sarstedt, Germany) and cultured overnight to obtain seed cultures. These seed cultures were then inoculated into 1 ml glc120 medium in a 24-well plate (Sarstedt) at an initial OD_600_ of 0.05. To minimize edge effects caused by evaporation and potential variation in oxygen availability [41], the first and last rows of all 24-well plates were filled with sterile glc120 medium and used as cell-free humidifiers; only the remaining inner rows were used for cultivation of yeast strains. A plate seal (Bio-Rad) was applied to prevent medium evaporation. Cultivations were carried out at 30°C and 900 rpm using a microplate rotator (Heidolph). After 60 h of cultivation, cells were harvested for analysis, as described in the “β-galactosidase activity measurement” section.

All strains used for RNA sequencing, fluorescence intensity measurement, and β-galactosidase activity measurement were cultivated in biological triplicates. All subsequent analyses were also performed in biological triplicates.

### RNA sequencing sample collection

Biomass were collected into chilled 50-ml Falcon tubes filled with 35 ml crushed ice. Samples were centrifuged for 4 min at 3000 × *g* and 4°C. Cell pellets were then washed once with 1 ml of chilled ultrapure water (18.2 MΩ·cm, Veolia Water Technologies, France), transferred into 1.5-ml Eppendorf tubes, and flash-frozen in liquid nitrogen. All cell pellets were subsequently sent to BGI genomics for RNA extraction and RNA sequencing.

### RNA extraction and sequencing

Total RNA was extracted from cell pellets using a phenol–chloroform-based method. RNA integrity was examined using a 2100 Bioanalyzer (Agilent Technologies, USA). RNA concentration was determined using a Qubit RNA HS Assay Kit (Thermo Fisher Scientific, USA).

RNA sequencing was performed using the DNBSEQ-G400 platform. Total RNA was first purified to enrich for polyadenylated RNAs using oligo (dT)-attached magnetic beads (NEB). The polyA-enriched RNA fraction was then fragmented and reverse transcribed into first-strand complementary DNA (cDNA) using random hexamer primers, followed by second-strand synthesis to generate double-stranded cDNA. The resulting fragments underwent end repair, 3′ adenylation, and adapter ligation. Adapter-ligated cDNA fragments were amplified by polymerase chain reaction (PCR) and purified using Ampure XP beads (Beckman Coulter, USA). Libraries were assessed for quality using a 2100 Bioanalyzer (Agilent Technologies). Circularization of double-stranded PCR products was performed to generate single-stranded circular DNA molecules, which were then amplified via rolling circle amplification using phi29 polymerase (NEB) to produce DNA nanoballs (DNBs). The DNBs were loaded onto a patterned nanoarray for sequencing by synthesis, generating paired-end 150-bp reads.

### RNA sequencing read alignment and processing

Sequencing reads were trimmed using trim_galore version 0.6.10 with cutadatp version 4.5 using parameters -j 4 -e 0.1 -q 20 -O 1 to remove adapters and low-quality reads and subsequently quality controlled using fastQC version 0.12.1. Reads were aligned using STAR version 2.7.10b. Gene counts were performed using featureCounts version 2.0.3 with parameters -p -s 2 -B --countReadPairs -T 4 to ensure strand-specific read counting of the paired-end reads and that only fragments where both ends are mapped were considered. Read-pairs instead of reads only were counted.

Reference genomes and genome annotation files were obtained from Ensembl Fungi for the *Y. lipolytica* W29 strain (GCA_001761485) [42].

### Identification of putative introns

After alignment, reads spanning disjoint segments in the genome were identified using the function summarizeJunctions from the R package GenomicAlignments, which returns a set of unstranded junction intervals ranging in size from 20 to 2000 nucleotides, hereafter referred to as putative introns. Only junctions with score 10 or higher across all samples were kept to consider as potential introns with at least 10 exon junction reads (EE) of support. Counting reads inside each putative intron was performed with the function summarizeOverlaps using mode=“IntersectionStrict”, singleEnd=FALSE, ignore.strand=TRUE, and inter.feature=FALSE to consider all reads strictly mapping to a putative intron interval. Reads overlapping the putative intron in any way (strictly or partially) were counted using summarizeOverlaps with mode=“Union”, singleEnd=FALSE, ignore.strand=TRUE, and inter.feature=FALSE. Exon–intron boundary reads were obtained from subtracting all putative intron reads with the strictly overlapping putative intron reads. For each intron, the mean splicing efficiency across all samples (SE) was used as a second threshold.

### Determination of intron splicing status in each condition

EE were taken as evidence of splicing, and half of the exon–intron boundary reads (EI) as evidence of intron retention. For each condition, introns with EE > 0 were classified as spliced. For EE = 0 and EI > 0, splicing status was tested against a background of pooled all EE > 0 conditions for the same intron using a chi-squared test, and classified as unspliced if significantly different (*P* < .05), or as unknown otherwise due to insufficient evidence for splicing. Introns with EE = 0 and EI = 0 were also classified as unknown due to insufficient expression. The number of conditions assigned to each splicing status for every intron is provided in Supplementary Table S4.

### Splicing efficiency estimation

For each intron, splicing efficiency was estimated based on sequencing reads mapped to exon–exon junctions (EE reads) and exon–intron boundaries (2× EI reads). As previously used in Wilhelm *et al*. [43], splicing efficiency was calculated using Equation (1). We acknowledge that this metric provides an approximation, as it does not account for differences in RNA degradation rates [44], which may affect the relative abundance of spliced and unspliced transcripts.


(1) \begin{eqnarray*}
&&\mathrm{Splicing}\ \mathrm{efficiency}= \\ && \frac{{\mathrm{number}\ \text{ of}\ \text{ EE}\ \mathrm{reads}}}{{\mathrm{number}\ \text{ of}\ \text{ EE}\ \mathrm{reads} + \mathrm{number}\ \text{ of}\ \text{ EI}\ \mathrm{reads}}}\ .
\end{eqnarray*}

### Mapping putative introns to genes

Annotated gene intervals were extracted from the gene annotation file downloaded from Ensembl Fungi for the *Y. lipolytica* W29 strain. These intervals were extended by 200 bp on either side in order to cover UTR regions. Introns intersecting these regions were attributed to the gene, and their distance to the annotated start and stop codons was computed.

For each intron overlapping more than one gene region, the proportion of the intron size falling within/close to each gene ORF was calculated, and the intron was then assigned to the gene with the highest overlap fraction/closest distance to the gene ORF.

Introns with a 5′ splice site located more than two times the length of the gene’s ORF upstream of the start codon (four introns in total), or with a 3′ splice site located more than two times the ORF length downstream of the stop codon (eight introns in total), were discarded.

### Gene ontology enrichment analysis

Gene ontology (GO) term annotations for the *Y. lipolytica* W29 genome were obtained from Lubuta *et al*. [45]. GO biological process enrichment analysis of intron-containing genes was performed using the clusterProfiler R package [46], applying a significance threshold of adjusted *P* < .05 (Benjamini–Hochberg method). All genes annotated with GO biological process terms in the genome were used as the background set. The code used for this analysis is provided in the “Data availability” section.

### Intron enrichment score calculation

For each gene region (r), the enrichment score was calculated as the number of observed introns divided by the number of all possible introns that could occur in that region, using Equation (2), considering only intron-containing genes. Possible introns were defined as all contiguous intervals at least 20 nucleotides in length whose start and end coordinates fall within the region boundaries, based on ORF coordinates with fixed 200-nucleotide 5′- and 3′UTRs, and were counted by enumerating all valid start–end pairs. Genome-wide enrichment scores were obtained by summing observed and possible counts across all intron-containing genes before computing the ratio:


(2) \begin{eqnarray*}
\mathrm{enrichment}\ \mathrm{score}{_\text{ r}} = \frac{{\mathrm{observed}\ \mathrm{introns}\ \text{ in}\ \text{ r}}}{{\mathrm{possible}\ \mathrm{introns}\ \text{ in}\ \text{ r}}}\ .
\end{eqnarray*}

### Intron potential branch point motif identification

To identify potential intron branch point positions, we developed a stepwise pattern search algorithm designed to locate the occurrence of a specific 6-nucleotide motif closest to the 3′ splice site within a given intronic sequence. The consensus branch point motif used as the reference was A–C–T–A–A–C [6], with nucleotide positions labeled 1 through 6. Position 5 was fixed as A in all pattern variants, corresponding to the putative branch point adenosine. A hierarchical mutation strategy was implemented to account for natural sequence variation, allowing single-nucleotide substitutions at only one position per search level. Mutation positions were evaluated in the following order of priority: 1 – 2 – 3 – 4 – 6. At each step, the base at the designated position was substituted with the three alternative nucleotides (excluding the reference), and all resulting pattern variants were scanned along the sequence. If one or more matches were found at a given level, the algorithm identified the last occurrence of any matching pattern (i.e. the one nearest to the 3′ splice site) and terminated the search. If no matches were identified, the search advanced to the next mutation level. Only one position was permitted to vary at each step; no multi-position substitutions were allowed. If no match was detected after all mutation levels were exhausted, the intron was classified as having no detectable branch point motif. The code used for the identification of potential branch point motifs is provided in the “Data availability” section.

### Consensus score calculation for intron core motifs

To quantify sequence conformity to consensus splicing motifs, we computed consensus scores for the 5′ splice site, 3′ splice site, and branch point using position weight matrices (PWMs). For each motif type, a position frequency matrix (PFM) was constructed from the corresponding intron motif sequences. PFMs were converted to log_2_-odd PWMs assuming a uniform background. Each sequence was scored, and scores were then normalized to a 0–1 range. For the branch point motif, only introns with a matched branch point sequence were used for PWM construction. Sequences without a detected branch point motif were assigned a default score of −1. The code used for this analysis is available in the “Data availability” section.

### Fluorescence analysis

Flow cytometry was conducted using an Attune NxT Flow Cytometer (Thermo Fisher Scientific) equipped with an Auto Sampler (Thermo Fisher Scientific). *Yarrowia lipolytica* cultures were diluted 1:6 in focusing fluid (30 µl culture broth + 150 µl focusing fluid; Thermo Fisher Scientific) and analyzed at a constant flow rate of 200 µl/min. At least 10 000 events per sample were collected. Single cells were identified by gating on forward scatter height versus area (FSC-H versus FSC-A). *hrGFP* fluorescence was detected using the BL1-A channel (excitation: 488 nm; emission: 574 nm; bandwidth: 20 nm) and normalized to FSC-A to account for cell size. Median fluorescence intensity was calculated for each population and used as the representative value per strain. Data processing and gating were performed in FlowJo v10.10.0, using the background strain Y09 as reference.

### Linear regression model development

To investigate the relationship between intron features and gene expression levels of single-intron genes or the fluorescence intensities of *hrGFP* strains, several linear regression models with an identity link function were developed and evaluated using cross-validation. Each model used intron features as predictors of expression outcomes. All intron features were standardized (*z*-score normalization) prior to model fitting. Model performance was evaluated by calculating the Pearson correlation coefficient between predicted and observed values. The code used for this analysis is provided in the “Data availability” section.

### β-galactosidase activity measurement

β-Galactosidase activity was measured using an Yeast β-Galactosidase Assay Kit (Thermo Fisher Scientific, Cat. No. 75768), following the microcentrifuge tube protocol. Briefly, 60-h cultures were mixed with a working solution containing Y-PER reagent and assay buffer, and the reaction was stopped upon color development. Cell debris was removed by centrifugation and absorbance of the supernatant was measured at 420 nm. Detailed procedures are provided in the manufacturer’s protocol.

### Statistical analysis

All statistical analyses were performed in R (version 4.2.2). Comparisons between two independent groups used two-tailed Student’s *t*-tests or Welch’s *t*-tests, depending on variance equality, or the Wilcoxon rank-sum test when parametric assumptions were not met. Paired non-parametric data were analyzed using the Wilcoxon signed-rank test. Multi-group non-parametric comparisons used the Kruskal–Wallis test. Associations between variables were assessed using Pearson or Spearman correlation coefficients, based on data distribution and linearity. Categorical data were analyzed using χ^2^ tests or Fisher’s exact tests. Multiple testing was controlled using the Benjamini–Hochberg FDR procedure where indicated. Statistical tests and *P*-values are reported in the figure legends and summarized in Supplementary Table S5.

## Results

### Carbon source and cell growth phase modulate splicing efficiency in *Y. lipolytica*

**Figure 1.**
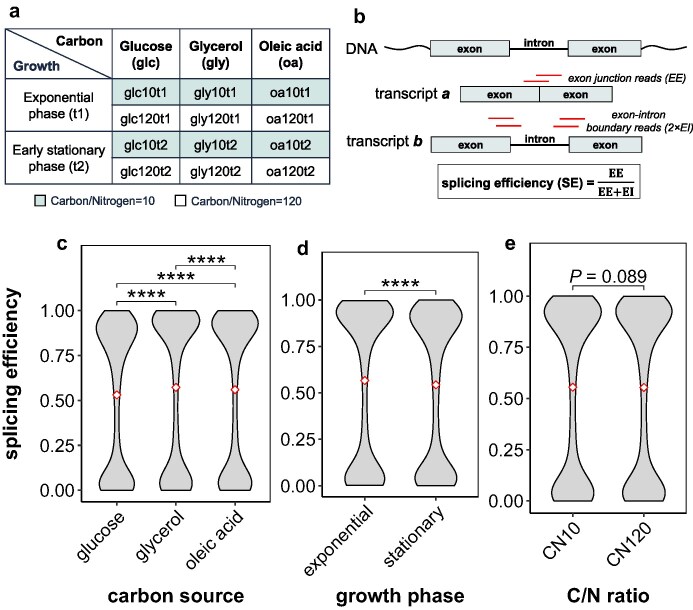
Splicing is influenced by carbon source and cell growth phase. (**a**) Overview of the 12 conditions used for RNA sequencing sample collection. Cells were grown and sampled on three different carbon sources [glucose (glc), glycerol (gly), and oleic acid (oa)], with varying carbon to nitrogen ratios and at two different growth phases. Sample collection and RNA sequencing were performed in biological triplicates for each condition. See the “Materials and methods” section for more details. (**b**) Schematic illustrating the procedures for intron identification and splicing efficiency estimation. Introns were identified based on RNA sequencing reads mapped to exon–exon junctions, and splicing efficiency was estimated by comparing reads mapped to exon–exon junctions with those mapped to exon–intron boundaries. EE, number of exon junction reads; EI, half the exon–intron boundary reads. (**c**) Splicing efficiency varies across carbon sources. For each intron subset, the mean splicing efficiency is indicated by a red diamond, as also shown in panels (d) and (e). (**d**) Splicing efficiency varies across cell growth phases. (**e**) Splicing efficiency remains constant across C/N ratios. In panels (c)–(e), a total of 2421 introns identified in this study are displayed. Intron splicing efficiency is represented as the mean splicing efficiency across conditions relevant to the indicated carbon source, growth phase, or C/N ratio. Statistical analyses were performed using a Wilcoxon signed-rank test (**** *P* < .0001).

The yeast *Y. lipolytica* has attracted significant interest due to its oleaginous nature and its capacity to metabolize a broad range of substrates. Intracellular lipid production reaches peak productivity during stationary phase when carbon is available but the supply of nitrogen in the growth medium is limited [47–49]. In industry, cells are therefore often first grown on rich medium with sufficient carbon and nitrogen to allow biomass accumulation during exponential growth, with a second stationary phase aimed at lipid accumulation by limiting the nitrogen supply. We therefore focused on three industrially relevant, key parameters—carbon source, carbon-to-nitrogen ratio, and growth phase—to characterize the intron landscape (Fig. 1a).

Introns are typically annotated from transcriptome data by identifying RNA-seq reads that align to two distinct but neighboring genomic regions, suggesting that they span exon–exon junctions formed after splicing (EE). However, some of these so-called “split reads” can represent false positives, arising from mapping artifacts in repetitive or low-complexity regions or from experimental artifacts such as reverse transcriptase template switching during cDNA synthesis in the RNA-seq procedure. To minimize such artifacts, a minimum EE-read threshold is often applied [50, 51]. In this study, we required at least 10 total EE reads across all samples as a baseline filter. Even in highly expressed genes, splicing leakage or the use of cryptic splice sites can also generate low-frequency junctions that mimic canonical introns [52, 53]. We therefore combined the EE threshold with a minimum splicing efficiency (SE) requirement to identify consistently spliced introns (Fig. 1b). Following Wilhelm *et al*. [43], splicing efficiency was defined as the ratio of reads supporting an exon–exon junction to all exon–exon junction reads plus EI overlapping the junction sites (Fig. 1b). Splicing efficiency reflects splicing rates; the faster the precursor RNA is spliced, the lower the relative abundance of EI reads compared to EE reads. However, it is important to note that the splicing efficiency calculated using steady-state unlabeled RNA-seq data is also influenced by mature RNA degradation because the faster the mature RNA is degraded, the lower the relative abundance of EE reads [44]. We evaluated three SE cutoff values (SE > 0.0001, SE > 0.001, and SE > 0.01) and compared the resulting intron and intron-containing gene counts (Supplementary Fig. S1a and b). About 500 well-expressed genes [10–1000 median transcripts per million (TPM) across the conditions] exhibit disproportionately low total EE (10–100), suggesting that such genes only contain introns that may be spliced out infrequently, for instance, via a cryptic splice site, or that the RNAs resulting from these splicing events are highly unstable (Supplementary Fig. S1c). The most stringent threshold, defined as “EE ≥ 10, SE > 0.01,” discarded those genes and yielded the strongest correlation between total EE and RNA abundance and was selected for downstream analysis. Using this threshold, we identified 2421 introns present in 18.1% (1430) of genes in *Y. lipolytica*, including 1302 previously unannotated introns and 479 intron-containing genes that had not been annotated as such before [6].

Splicing of the majority of introns (76.7%; 1857 out of 2421) was detected across all 12 conditions. Of the remaining 564 introns, splicing of 37 was not detected in 1 to 7 conditions, while the other 527 exhibited no detectable EE reads and either very low or undetectable EI reads in 1 to 10 conditions, likely due to low gene expression, making it impossible to assess their splicing status (for methodological details, see the “Determination of intron splicing status in each condition” section; for the intron list with associated splicing status annotations, see Supplementary Table S4).

Most intron-containing genes show some level of splicing across all 12 tested conditions, with slight variations in overall splicing efficiency (Fig. 1c–e). Among the 12 tested conditions, carbon source and growth phase significantly impacted overall splicing efficiency. Cells grown on glycerol exhibited the highest overall splicing efficiency, followed by oleic acid, with glucose showing the lowest (Fig. 1c). Correspondingly, 81.4% and 79.7% of introns showed higher mean splicing efficiency in glycerol and oleic acid than in glucose, respectively. These fractions increased, respectively, to 91.2% and 89.4% when restricting the analysis to introns with mean splicing efficiency of at least 0.5 under glucose (Supplementary Fig. S2a and b). Similarly, 70.2% of introns displayed higher splicing efficiency in glycerol than in oleic acid, rising to 79.6% among introns with mean efficiency of at least 0.5 under oleic acid (Supplementary Fig. S2c). Splicing efficiency was also generally higher in exponential than in early stationary phase (Fig. 1d). In total, 70.5% of introns exhibited higher mean efficiency in exponential phase, increasing to 84.2% among those with mean efficiency of at least 0.5 under early stationary phase (Supplementary Fig. S2d). In contrast, carbon-to-nitrogen ratio shows a more moderate effect on splicing efficiency with 47.1% of introns showing higher efficiency in CN10 than in CN120 (Fig. 1e and Supplementary Fig. S2e).

### Intron-containing genes display high and stable gene expression across conditions

**Figure 2.**
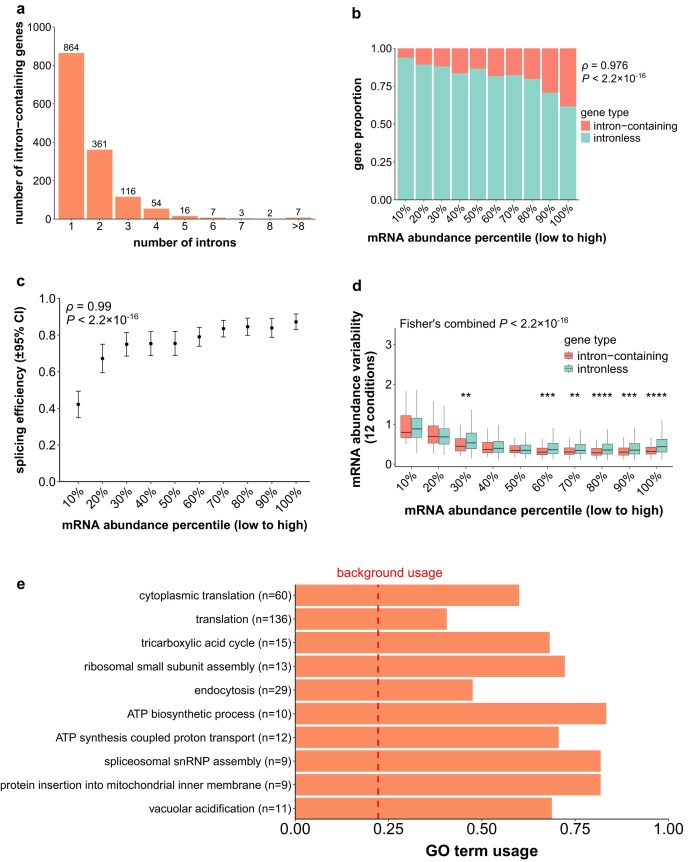
Genes containing introns display high and stable gene expression. (**a**) Number of distinct introns per intron-containing gene. Numbers above bars indicate corresponding gene counts. (**b**) Intron-containing genes exhibit higher mRNA abundance than intronless genes. A total of 7898 genes were analyzed. Genes are ranked by their median mRNA abundance (TPM) across 12 conditions and grouped into 10 expression deciles. Bars show intron-containing and intronless gene proportions by color. Statistical analysis was performed using Spearman correlation (*ρ* = 0.976, *P* < 2.2 × 10^−16^). (**c**) In single-intron genes, mRNA abundance correlates strongly with the corresponding intron splicing efficiency. A total of 864 single-intron genes were analyzed. Genes are ranked by their median TPM across 12 conditions and grouped into 10 expression deciles. Statistical analysis was performed using Spearman correlation (*ρ* = 0.99, *P* < 2.2 × 10^−16^). Error bars represent 95% confidence interval (CI) of splicing efficiency by decile. (**d**) Intron-containing genes exhibit more uniform mRNA abundance across conditions compared to intronless genes. Genes are ranked by their median TPM across 12 conditions and grouped into 10 expression deciles. A Wilcoxon rank-sum test was performed for each decile (**P < .01, ***P < .001, ****P < .0001), and overall significance across the 10 expression bins was evaluated using Fisher’s combined probability test applied to the individual Wilcoxon *P*-values. (**e**) Intron-containing genes are enriched in cellular translation and respiration-related processes. The top 10 GO biological process terms with the lowest adjusted *P*-values for enrichment of intron-containing genes against the genomic background are shown. GO term usage is defined as the ratio between the number of intron-containing genes within a GO term and the total number of genes associated with that term. The red dashed line (background usage) represents the fraction of intron-containing genes across all genes annotated to GO biological process terms. The *n* after each GO term indicates the number of intron-containing genes belonging to this GO term. Statistical analysis was performed using Fisher’s exact test (adjusted *P*-value <.05).

Among the 1430 identified intron-containing genes, the majority (60.4%; 864 genes) contain a single intron, while 25.2% (361 genes) harbor two introns (Fig. 2a). The gene *YALI1_A06530g* has the highest number of introns [43]. It is a putative gene without annotation [42], and its highly repetitive coding sequence may have caused read-mapping artifacts, leading to the large number of potential introns detected. Of the 566 genes with multiple introns, 481 exhibit alternative splice sites, meaning that two or more introns overlap or share partial sequences (Supplementary Fig. S3).

mRNA abundance varied widely among genes, with TPM values ranging from <0.01 to over 10 000 (for detailed mRNA abundance across 12 conditions, see Source data). We find that as mRNA abundance increased, the proportion of intron-containing genes also increased—a trend consistent with observations in other organisms [54, 55] (Spearman correlation *ρ* = 0.976, *P* < 2.2 × 10^−16^; Fig. 2b). Notably, the only two genes with median TPM values exceeding 10 000 both contain introns. This could reflect that higher expressed genes are more stable and, not exclusively, that genes with higher expression are more efficiently spliced. To investigate the relationship between splicing and expression, the 864 single-intron genes were ranked by median mRNA abundance and divided into 10 deciles. We found that splicing efficiency increased with expression levels (Spearman correlation *ρ* = 0.99 with expression level decile, *P* < 2.2 × 10^−16^; Fig. 2c). Moreover, we assessed whether introns were associated with mRNA expression variability across the 12 conditions, as measured by the coefficient of variation (CV), controlling for mRNA abundance deciles. This stratified analysis was an important control because expression level can bias intron detection and because the CV scales non-linearly with the expected RNA-seq read counts [56]. The analysis revealed that intron-containing genes generally exhibit lower expression variability compared to intronless genes (Fisher’s combined *P* < 2.2 × 10^−16^; Fig. 2d). This suggests a potential link between intron presence and gene expression stability, with genes containing introns being more constitutively expressed across different conditions and growth phases.

GO biological process enrichment analysis indicated that introns are enriched in housekeeping genes. For example, an important functional category of genes that is significantly enriched for introns is involved in translation-related processes, including cytoplasmic translation, general translation, and ribosomal small subunit assembly (Fisher’s exact test, adjusted *P* < .05; Fig. 2e and Supplementary Table S6). Among the intron-containing genes, 136 are related to translation, including those involved in large and small ribosomal subunit maturation and assembly, such as *YALI1_A09798g* (*RPL32*), *YALI1_E37930g* (*RPL37*), *YALI1_F27175g* (*RPS11*), and *YALI1_F08431g* (*RPS12*). Additional intron-containing genes contribute to translational initiation, elongation, and fidelity regulation, including *YALI1_D32002g* (*CDC33*, encoding a translation initiation factor that binds the 5′ cap of mRNA) and *YALI1_C12642g* (*TEF1*, encoding elongation factor 1-α).

Intron-containing genes also show strong enrichment in cellular respiration pathways, such as the tricarboxylic acid (TCA) cycle, ATP biosynthesis, ATP synthesis-coupled proton transport, protein insertion into mitochondrial inner membrane, and vacuolar acidification (Fig. 2e and Supplementary Table S6). Out of the 22 genes annotated to the TCA cycle (GO:0 006099), 15 contain an intron. Similarly, 10 of the 12 genes involved in the ATP biosynthetic process (GO:0 006754) contain introns.

### Introns are enriched near the translational start codon

**Figure 3.**
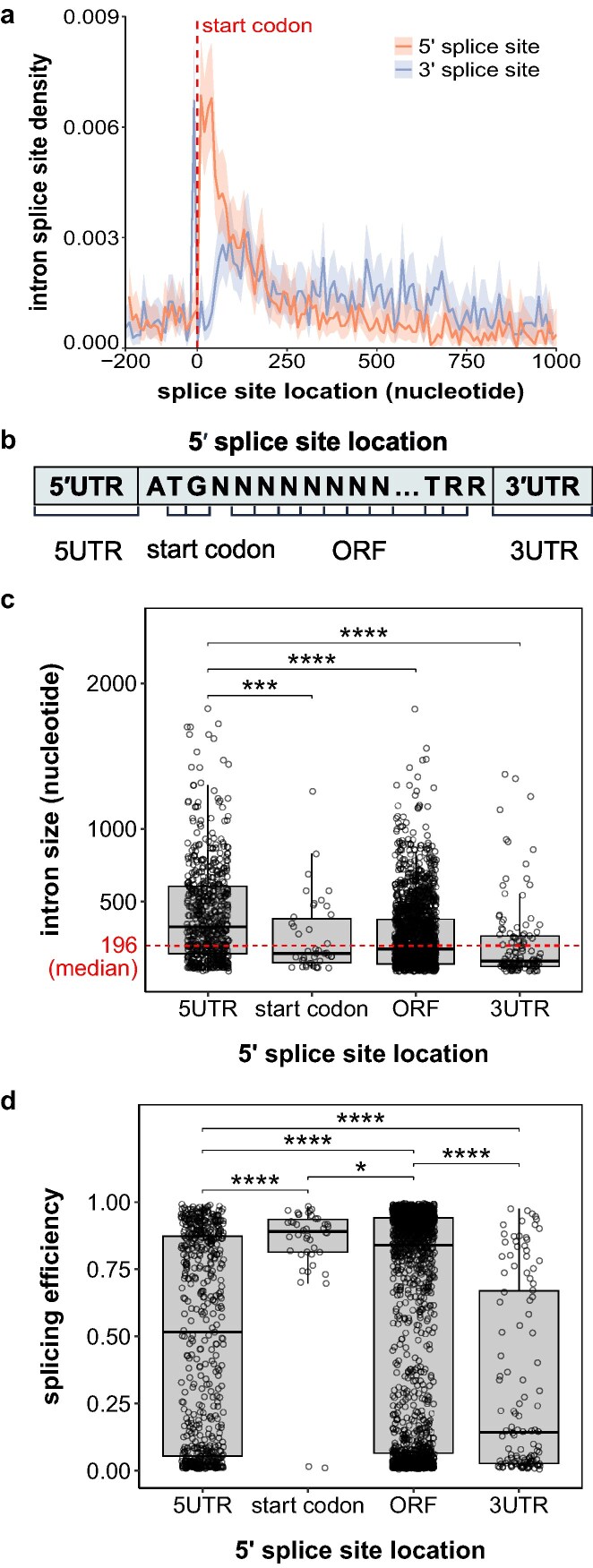
Intron location correlates with intron size and splicing efficiency. (**a**) Introns are primarily located around the start codon. The 5′/3′ splice site density is calculated as the ratio of the number of unique 5′/3′ splice sites to the overall number of unique nucleotides in each window across all intron-containing genes. A 10-nucleotide sliding window was used for analysis. Light-colored ribbons represent the 95% CI of the densities. The *x*-axis is centered at the start codon (0), with a scale from −200 to 1000 nucleotides based on intron distribution in most intron-containing genes. (**b**) Schematic illustrating the relative locations of introns within gene structures, corresponding to how 5′ splice site locations are classified in panels (c) and (d). The 5′ splice site location is defined as the position immediately following a nucleotide within the 5′UTR, ORF, or 3′UTR. The category “start codon” is treated separately from the “ORF” group and specifically refers to the 42 introns whose 5′ splice sites occur immediately after the start codon nucleotide (A, T, or G). “N” in the sequence represents any of the 4 nucleotides (A, T, C, or G); “R” denotes a purine (A or G). “TRR” corresponds to stop codons such as TAG, TGA, or TGG. (**c**) 5′UTR introns tend to be longer than introns in other regions. Intron counts per box (left to right): 561, 42, 1700, 118. Across all introns, the median size was 196 nucleotides. Statistical analysis was performed using a two-tailed Welch’s *t*-test (unequal variance) (***P < .001, ****P < .0001). (**d**) Start codon introns and ORF introns tend to have higher splicing efficiency than UTR introns. Intron counts per box are identical to panel (c). Intron splicing efficiency is represented as the mean splicing efficiency across all conditions where the intron is spliced. Statistical analysis was performed using a Wilcoxon rank-sum test (*P < .05, ****P < .0001).

Introns are not evenly distributed across genes, with both 5′ and 3′ splice site densities peaking near the translation start codon. Since high-confidence UTR annotations are lacking for *Y. lipolytica*, we considered the 200 nucleotides upstream of the start codon as the 5′UTR and the 200 nucleotides downstream of the stop codon as the 3′UTR. The highest density of 5′ splice sites occurs within the first 10 nucleotides of the ORF, while the highest density of 3′ splice sites is found within the last 10 nucleotides of the 5′UTR (Fig. 3a), suggesting that intron location is not random, but instead shaped by some form of selective pressure [57]. Interestingly, some introns originating in the 5′UTR extend into the ORF or even into the 3′UTR, which implies that splicing of these introns could have strong effects on the expressed protein (Supplementary Fig. S4a).

To quantify regional enrichment, we defined an “intron enrichment score” by normalizing the observed intron count in a given region to the total number of splicing possibilities within that region (i.e. taking into account all possible pairs of 5′ and 3′ splice sites whose corresponding sequence, including both splice sites, is at least 20 nucleotides long; see the “Intron enrichment score calculation” section. Consistent with the 5′ splice site density peak, introns that start within the first 10 nucleotides of the ORF and end within the ORF (N10–ORF) show a 10.2-fold enrichment relative to the overall intron enrichment score (Supplementary Fig. S4b). Furthermore, we observed strong enrichment for introns that are entirely embedded within the 5′UTR or 3′UTR, with 8.8-fold and 5.9-fold increases in enrichment score, respectively, potentially reflecting selective pressure to preserve ORF integrity (Supplementary Fig. S4c).

All introns were categorized into four groups based on their 5′ splice site location; “5UTR,” “start codon,” “ORF,” and “3UTR” (Fig. 3b). Intron size varies widely, from 20 to over 1000 nucleotides, with a median of 196 nucleotides. Introns with 5′ splice sites located within the 5′UTR are significantly longer (median = 325 nucleotides) compared to those with 5′ splice sites within the start codon, ORF, or 3′UTR (Fig. 3c). Interestingly, intron location correlates with splicing efficiency. Introns located near the start codon show the highest splicing efficiencies, and those within the 3′UTR show the lowest splicing efficiencies (Fig. 3d). This correlation also holds for gene expression (Supplementary Fig. S5a). Genes containing introns in the start codon region show the highest expression and lowest expression variability across 12 conditions, whereas those with introns in their 3′UTR exhibit the lowest mRNA abundance and greatest variability (Supplementary Fig. S5a and b). Overall, these patterns suggest that intron location is connected with intron size, splicing efficiency, and even gene expression.

Intron retention, a form of alternative splicing, has substantial regulatory implications by generating transcripts that are retained in the nucleus, degraded via the nonsense-mediated decay pathway, or subject to uORF-mediated translational repression [58, 59]. Notably, these effects are closely tied to intron location. We found that 68.3% (383 out of 561) of 5′UTR introns contain an upstream start codon followed by an in-frame stop codon, potentially forming uORFs that may repress translation of the main ORF. Additionally, 82.0% (1426 out of 1738) of ORF introns harbor premature stop codons, possibly targeting the transcripts for nonsense-mediated decay. These findings suggest that intron retention generally reduces gene and protein expression. Only a small fraction of retained introns—11 out of 561 in the 5′UTR and 143 out of 1738 in the ORF—preserve the original reading frame, thereby offering the potential to form variants of the same protein, although this does not imply that all variants are functional. Moreover, 109 of the 561 retained 5′UTR introns do not introduce upstream start codons. In these cases, intron retention might only have minimal consequences for gene expression (Supplementary Fig. S6).

### Intron core motifs and sequence features influence splicing and gene expression

**Figure 4.**
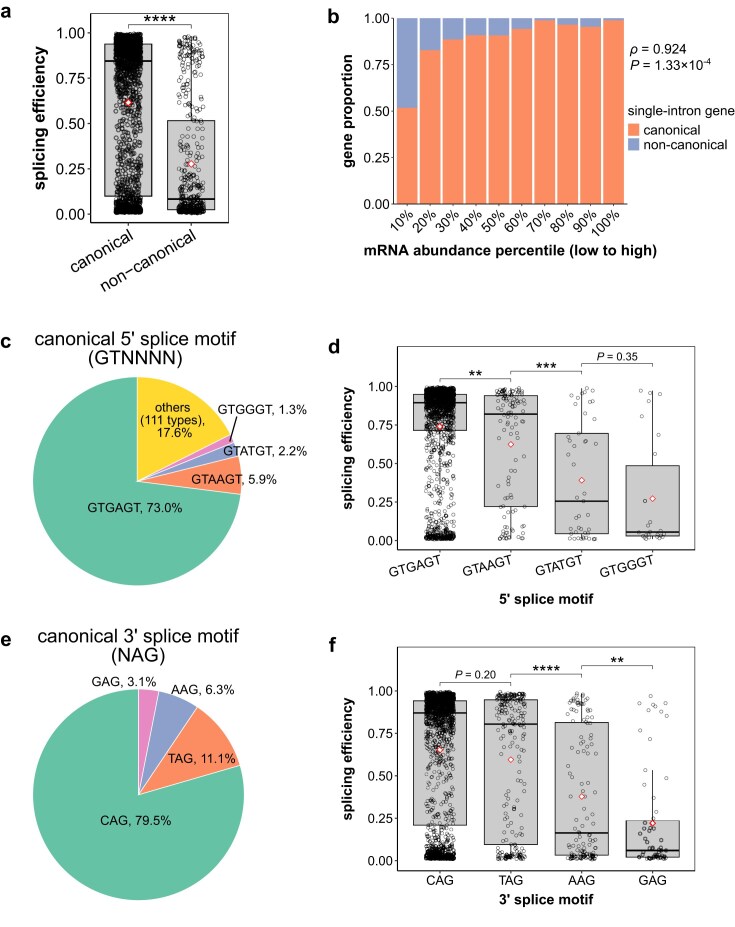
Splicing is dependent on the intron splice motifs. (**a**) Canonical introns tend to have higher splicing efficiency than non-canonical ones. Canonical introns: 2012; non-canonical introns: 409. For each intron subset, the mean splicing efficiency is indicated by a red diamond, as also shown in panels (d) and (f). (**b**) Single-intron genes with canonical introns exhibit higher mRNA abundance than those with non-canonical introns. A total of 864 single-intron genes were analyzed. Genes are ranked by their median TPM across 12 conditions and grouped into 10 expression deciles. Statistical analysis was performed using Spearman correlation (*ρ* = 0.924, *P* = 1.33 × 10^−4^). (**c**) The diversity of 5′ splice motif of canonical introns. (**d**) 5′ splice motif influences the splicing efficiency. Intron counts per box (left to right): 1468, 118, 45, 26. (**e**) The diversity of 3′ splice motif of canonical introns. (**f**) 3′ splice motif influences the splicing efficiency. Intron counts per box (left to right): 1599, 223, 127, 63. In panels (a), (d), and (f), intron splicing efficiency is represented as the mean splicing efficiency across all conditions. Statistical analyses were performed using a Wilcoxon rank-sum test (**P < .01, ***P < .001, ****P < .0001).

83.1% (2012) of the 2421 introns are canonical, defined by having a “GT” sequence at the 5′ splice site and an “AG” at the 3′ splice site. Canonical introns display significantly higher splicing efficiency than non-canonical introns, with a mean splicing efficiency of 0.61 compared with 0.28 for non-canonical introns (Wilcoxon rank-sum test, *P* < 2.2 × 10^−16^; Fig. 4a). Moreover, genes harboring canonical introns show higher expression levels than those containing non-canonical introns (Fig. 4b).

These findings motivated a deeper investigation into the sequence diversity of splice motifs within canonical introns. Analysis of the first six intronic nucleotides at the 5′ splice site revealed 115 distinct motifs. The most prevalent motif is GTGAGT, accounting for 73.0% of canonical introns. This is a notable departure from the predominant motif GTATGT observed in most of hemiascomycetous yeasts such as *S. cerevisiae* and *Kluyveromyces marxianus* [60, 61], which can be mechanistically explained by base-pair complementarity with *Y. lipolytica* U1 RNAs [6]. The next three most frequent motifs are GTAAGT (5.9%), GTATGT (2.2%), and GTGGGT (1.3%) (Fig. 4c). Introns containing the GTGAGT motif exhibit the highest splicing efficiencies and generally, introns with more frequent 5′ splice motifs are associated with higher splicing efficiencies (Fig. 4d). Similarly, the most common 3′ splice motif is CAG, present in 79.5% of introns, followed by TAG (11.1%), AAG (6.3%), and GAG (3.1%) (Fig. 4e). Introns containing CAG as 3′ splice motif have the highest mean splicing efficiency (0.65), followed by those with TAG (0.60) (Fig. 4f).

When looking at the combination of 5′ and 3′ splice site sequences, introns containing more common motifs at both the 5′ and 3′ splice sites tend to exhibit higher splicing efficiencies than those that do not (Fig. 4d and f). Comparing splicing efficiency across introns grouped by combinations of 5′ and 3′ splice motifs showed that those with GTGAGT-CAG or GTGAGT-TAG combination display the highest splicing efficiencies, indicating that these sequences might represent the optimal choice for efficient splicing in synthetic designs (Supplementary Fig. S7a; see also further).

Besides 5′ and 3′ splice motifs, the intron branch point is also an important motif influencing intron splicing [62]. Branch points typically show greater sequence flexibility compared to splice sites, and individual introns may contain multiple potential branch points [63]. Since ACTAAC is the predominant branch point motif across many yeast species and usually resides near the 3′ splice site [6, 60], we investigated each intron sequence for the occurrence of ACTAAC directly upstream of the 3′ splice site to designate as the branch point. If ACTAAC was absent, the nearest 6-nucleotide sequence differing by only 1 nucleotide (with the 5th nucleotide always remaining adenine) was selected as an alternative branch point motif. We identified 16 distinct potential branch point motifs within 1950 canonical introns. The most frequent motif is ACTAAC, present in 67.5% of introns, and it is associated with a high mean splicing efficiency (0.65) (Supplementary Fig. S7b). Other common motifs include GCTAAC (present in 14.7% of introns, with a mean splicing efficiency of 0.62), TCTAAC (7.5%, 0.63), ATTAAC (2.6%, 0.50), CCTAAC (1.9%, 0.61), and ACTAAT (1.5%, 0.54).

Distances from branch points to 3′ splice sites are generally short, with 94.4% of introns showing a distance of no more than 10 nucleotides. This is notably shorter than in most other organisms [64] and may contribute to stabilizing introns when excised in linear form [16]. Remarkably, 72.5% of introns only contain a single nucleotide between these motifs, and two introns even exhibit a 1-nucleotide overlap (distance of −1), sharing a cytosine nucleotide (Supplementary Fig. S7c). The distance also shows a significant correlation with splicing efficiency, as all mean splicing efficiencies are greater than 0.41 and mostly above 0.52 when the distance ranges from 0 to 5 nucleotides, whereas the mean splicing efficiency decreases to 0.23 when the distance exceeds 5 nucleotides (Supplementary Fig. S7d).

Furthermore, intron GC content correlates with splicing efficiency [65]. Introns with intermediate GC content (0.35–0.55) consistently show high mean splicing efficiencies (higher than 0.57). In contrast, those with either low (<0.35) or high (>0.55) GC content exhibit substantially lower splicing efficiencies, with mean values typically below 0.44 (Supplementary Fig. S7e). Intron size also shows a weak correlation with splicing efficiency. Introns 100–200, 200–300, or 700–1826 nucleotides in length exhibit lower splicing efficiency than those in the other size ranges (Supplementary Fig. S7f).

Overall, all six examined intron sequence features (5′ splice motif, 3′ splice motif, branch point motif, distance from branch point to 3′ splice site, GC content, intron size) correlate with splicing efficiency (Fig. 4 and Supplementary Fig. S7). As each optimal feature type, defined as the feature type exhibiting the highest splicing efficiency, consistently shows a bimodal distribution of splicing efficiency, we further examined the correlation between splicing efficiency and the number of optimal sequence features within introns exhibiting extreme splicing efficiencies (below 0.1 or above 0.9). Among the 2421 identified introns, 723 show mean splicing efficiency below 0.1, while 802 show mean splicing efficiency above 0.9 (Supplementary Fig. S8). In the low splicing-efficiency group, 695 (96.1%) contain at least one optimal sequence feature, 706 (97.6%) contain at least one non-optimal sequence feature, and 649 (89.8%) contain two or more non-optimal features. In contrast, in the high splicing-efficiency group, all introns contain at least one optimal feature, 726 (90.5%) contain at least four optimal features, and 266 (33.2%) contain all six optimal features. These findings suggest that splicing efficiency is determined by the combined influence of multiple intron sequence features, rather than any single element alone.

Given the crucial role of introns in gene regulation, we investigated whether native gene expression could be predicted based on the six examined intron sequence features, despite each gene being embedded within distinct genomic contexts and subject to diverse regulatory mechanisms. To compare the predictive power of the six individual features that were included in our model for the prediction of mRNA abundance across 864 single-intron genes, we applied linear regression models using identical 10-fold cross-validation. All six features exhibit positive correlations with gene expression, with the three core splicing sequences, namely 5′ splice site, branch point, and 3′ splice site, demonstrating relatively strong predictive power, highlighting the importance of the splicing process in regulating gene expression (Supplementary Fig. S9a). Furthermore, a linear regression model was constructed using the same cross-validation strategy, based on a combination of six sequence features (Supplementary Fig. S9b). The model demonstrated relatively strong predictive power, achieving a Pearson correlation coefficient of 0.40 (*P* < 2.2 × 10^−16^). These results show that intron features can be used to predict *in situ* gene expression levels, despite variation in promoters, UTRs, terminators, and other regulatory elements.

### Intron-mediated gene regulation is tunable and predictable

**Figure 5.**
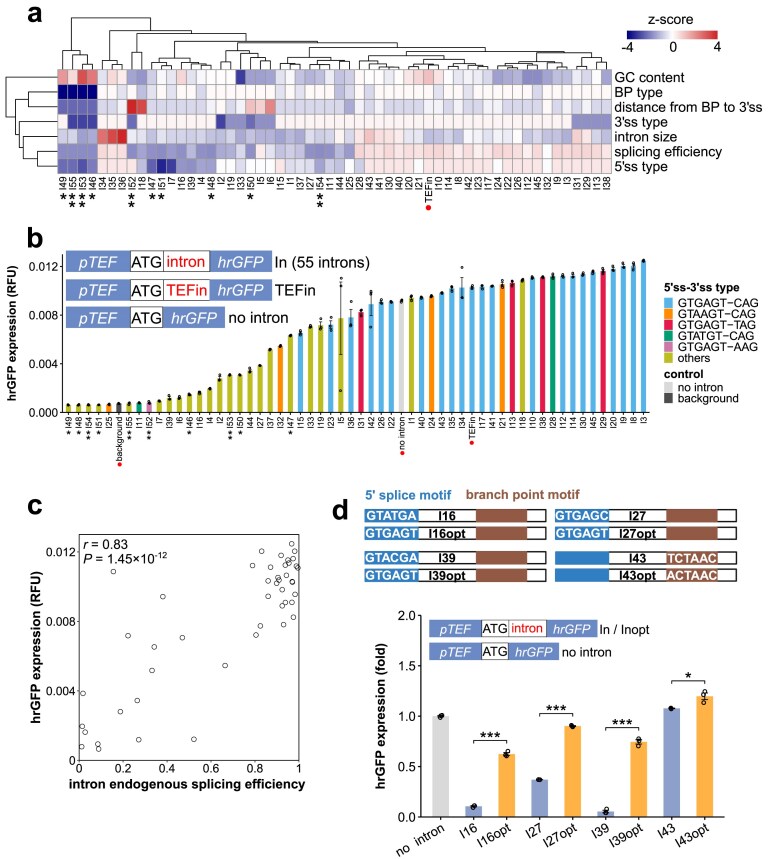
Intron-mediated gene regulation is tunable and predictable. (**a**) 55 selected introns display large diversity in seven key intron features. The commonly used TEF intron (TEFin) is included as a reference and marked with a red dot. Six introns labeled with * belong to the “EE ≥ 10, SE > 0.001” threshold group, and four introns labeled with ** belong to the “EE ≥ 10, SE > 0.0001” threshold group, as defined in Supplementary Fig. S1. Intron splicing efficiency is represented as the endogenous mean splicing efficiency across all conditions. The 5′ss type, 3′ss type, and BP (branch point) type are represented by their respective consensus scores. All data are normalized as *z*-scores. The method used for calculating consensus score is described in the “Materials and methods” section. EE, number of exon junction reads; SE, spicing efficiency; ss, splice site. (**b**) The selected 55 introns enable a 199-fold range of fluorescence intensity. The background (strain Y09, no *hrGFP* expression cassette), no intron (*pTEF* only), and TEFin (*pTEF* + *TEF* intron) were evaluated as well and marked with red dots. Introns labeled with * or ** correspond to the threshold groups described in panel (a). Every expression cassette was integrated into the genome in single copy. The glc120 medium was used, and each strain was evaluated in biological triplicates (*n* = 3). The bars indicate the mean fluorescence, with error bars representing the corresponding standard errors. Each bar includes three dots, representing the fluorescence values of the biological triplicates. The 5′ss–3′ss motifs of each intron are shown in color. RFU, relative fluorescence units. (**c**) Endogenous intron splicing efficiency correlates strongly with *hrGFP* expression. Forty-six strains containing selected introns from the “EE ≥ 10, SE > 0.01” group or the *TEF* intron are included. The correlation was assessed using Pearson’s method. Endogenous splicing efficiency is represented as the endogenous mean splicing efficiency across 12 conditions. (**d**) Single-feature optimization of the 5′ splice motif or branch point motif increases intron-mediated gene expression. Top, schematic representation of the 5′ splice motifs of the natural introns I16, I27, and I39 and their corresponding optimized variants, I16opt, I27opt, and I39opt, as well as the branch point motif of the natural intron I43 and its optimized variant, I43opt. All other nucleotides are identical between each natural intron and its corresponding optimized variant. Bottom, bar plot showing that optimized introns increase hrGFP expression compared with their corresponding natural introns. Each expression cassette was integrated into the genome as a single copy. Cells were grown in glc120 medium, and each strain was evaluated in biological triplicate (*n* = 3). hrGFP expression levels were normalized to the no-intron reference strain. Bars indicate mean fluorescence and error bars represent standard errors of the mean. Dots indicate fluorescence values from individual biological replicates. Statistical significance was assessed using a two-tailed Student’s *t*-test (*P < .05, ***P < .001). The top part of panel (d) was created in BioRender. Verstrepen, K. (2026) https://BioRender.com/9h4vm89.

The capacity to predict gene expression from intronic features could be due to a mechanistic role of splicing in enhancing expression, e.g. via enhanced transcription or RNA stability, but could also be confounded by other sequence elements that are typically associated with highly expressed genes such as strong promoters or stabilizing RNA sequence elements including optimal codons. To experimentally validate an actual role of intronic sequence on gene expression in *Y. lipolytica*, we selected 45 introns to represent the majority of the diversity across the feature space and tested their potential to modulate gene expression (Fig. 5a and Supplementary Table S7). To extend the range of intron splicing efficiency, we included six additional introns with endogenous mean splicing efficiencies between 0.1% and 1% from the “EE ≥ 10, SE > 0.001” dataset, and four introns with endogenous mean splicing efficiencies between 0.01% and 0.1% from the “EE ≥ 10, SE > 0.0001” dataset (Fig. 5a and Supplementary Table S7). The widely used *TEF* intron (TEFin), commonly paired with the *TEF* promoter to enhance gene expression in genetic engineering applications, was included as a reference.

These 55 introns were evaluated within an *hrGFP* expression cassette driven by *pTEF* (comprising the *TEF* promoter and 5′UTR), integrated at the IntC2 genomic locus that is often used to integrate heterologous constructs [66]. Since TEFin naturally resides downstream of the start codon, and our previous observations showed that introns in the start codon region exhibit high splicing efficiencies and high mRNA abundance, all selected introns were placed immediately downstream of the start codon. For comparison, control constructs containing only *pTEF* (no intron) and *pTEFin* (*pTEF* + TEFin) were also included in the assay (Fig. 5b).

Of the 45 strains carrying introns with endogenous mean splicing efficiencies above 1%, 44 showed significant *hrGFP* expression (fluorescence above the background strain; one-tail Student’s *t*-test, *P* < .05). In comparison, only 4 out of 10 strains carrying introns with endogenous mean splicing efficiencies between 0.01% and 1% showed measurable expression of *hrGFP*. Among these 4 strains, 2 exhibited the highest splicing efficiency (>0.7%) of the 10 strains, while the other 2 (I46 and I53) have splicing efficiencies of 0.2% and 0.05%. Overall, our results demonstrate that intron endogenous splicing efficiency may influence *hrGFP* expression, with very low splicing efficiencies leading to non-measurable expression. Specifically, in our test setup, introns with endogenous mean splicing efficiencies above 0.7% generally showed measurable gene expression (46 out of 47 introns; 97.9%).

For the 46 strains with significant *hrGFP* expression and splicing efficiencies above 0.7%, the expression range varied widely, from 0.7% to 139.0% relative to the no-intron *TEF* reference, representing a 199-fold range (Fig. 5b). The construct with the highest *hrGFP* expression, I3, contains intron id4686, which harbors the predominant splice site motif GTGAGT-CAG and branch point motif ACTAAC. This intron originates from *MFE2* (*YALI1_E18441g*), a gene encoding a peroxisomal multifunctional β-oxidation enzyme required for fatty acid utilization [67]. Notably, 18 out of the 23 constructs exceeding the expression levels of the reference *TEF* construct contained introns with the most optimal splice site motif GTGAGT-CAG or GTGAGT-TAG, as shown in Supplementary Fig. S7a.

As higher endogenous intron splicing efficiencies generally led to measurable *hrGFP* expression, we next examined whether endogenous splicing efficiency correlates with *hrGFP* levels within the set of 46 introns exhibiting high endogenous splicing efficiencies (endogenous mean splicing efficiency >1%; 45 selected introns plus the *TEF* intron). A linear regression analysis revealed a strong positive correlation between endogenous splicing efficiency and *hrGFP* expression, with a Pearson correlation coefficient of 0.83 (*P* = 1.45 × 10^−12^; Fig. 5c).

We additionally assessed the correlation between the six examined intron sequence features and *hrGFP* expression. To do so, we applied linear regression models with four-fold cross-validation to the set of 46 introns. Among the individual features, the 5′ splice site demonstrated the strongest positive correlation and predictive performance, whereas both the 3′ splice site and distance from the branch point to the 3′ splice site showed negative correlations (Supplementary Fig. S10a). When all six features were combined, the model yielded a Pearson correlation coefficient of 0.44 (*P* = 2.23 × 10^−3^; Supplementary Fig. S10b).

To further investigate the relationship between intron splicing and gene expression, we optimized selected non-optimal splice motif-related features of four natural introns and evaluated their effects using the *hrGFP* expression system. Specifically, we selected three introns, I16, I27, and I39, each containing a non-optimal 5′ splice motif but optimal profiles for the other five features, and one intron, I43, which contains a non-optimal branch point motif but optimal profiles for the other five features. Among the 55 natural introns tested in Fig. 5b, no intron contained a non-optimal 3′ splice motif while retaining optimal profiles for the other five features, suggesting that the 3′ splice motif may co-vary with additional intronic features. We then optimized the 5′ splice motifs of I16, I27, and I39 to generate I16opt, I27opt, and I39opt, respectively, and the branch point motif of I43 to generate I43opt (Fig. 5d). These optimized introns showed *hrGFP* expression levels 5.88-, 2.43-, 13.67-, and 1.11-fold those of their corresponding natural introns, respectively (Fig. 5d), supporting an important role for intron splicing in intron-mediated gene regulation.

### Robustness of intron-mediated regulation across genomic contexts

To further examine intron-mediated regulation across genomic contexts, we repeated the reporter-based assay but altered both the expressed gene and the genomic location of the reporter construct, evaluating introns within a *lacZ* expression cassette integrated at the commonly used IntB locus [68].

**Figure 6.**
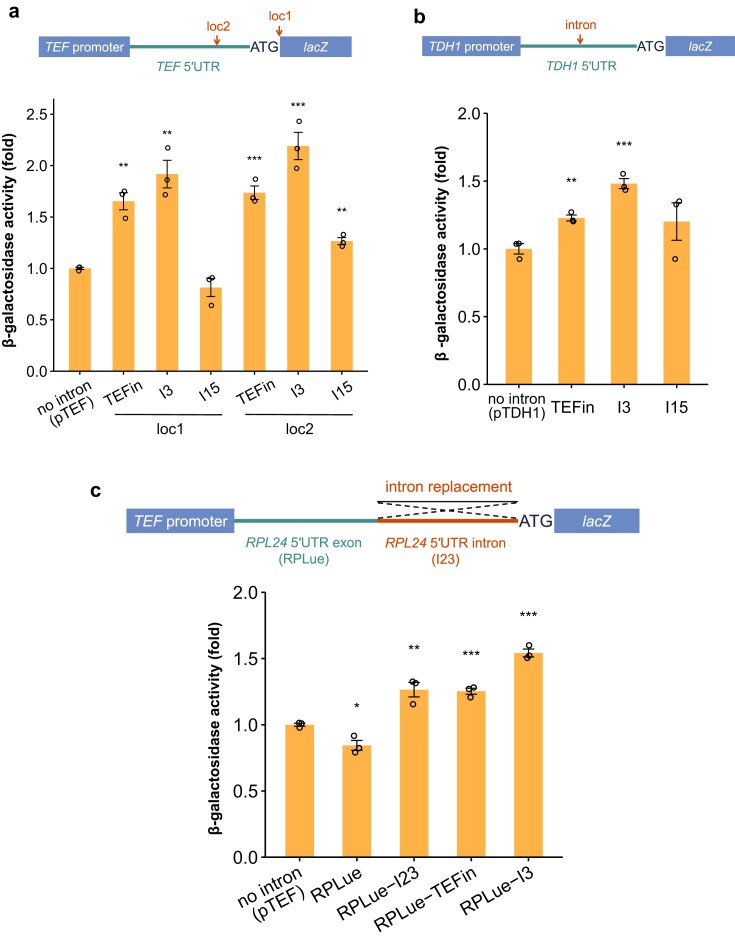
Introns can modulate protein expression across genomic contexts. (**a**) Introns can modulate protein expression. Different intron locations in the reporter *pTEF*-*lacZ* expression cassette were tested. The natural *TEF* intron location is loc1, loc2 is 11 nucleotides upstream the start codon. *TEF* intron, I3, and I15 were evaluated, with the no-intron construct (*pTEF*) as control. I3 showed the highest fluorescence in Fig. 5b, whereas I15 showed 68.7% of the fluorescence of the no-intron strain. (**b**) Introns regulate β-galactosidase activity in the *pTDH1*-*lacZ* expression cassette. *TEF* intron, I3, and I15 were evaluated, with the no-intron construct (*pTDH1*) as control. (**c**) Introns regulate β-galactosidase activity in the *TEF* promoter-*RPL24* 5′UTR-*lacZ* expression cassette. *TEF* intron, I3, and I23 were evaluated, with the no-intron construct (*pTEF*) as control. I23 showed 76.8% of the fluorescence of the no-intron strain in Fig. 5b. RPLue, *TEF* promoter-*RPL24* 5′UTR exon-*lacZ*; RPLue-intron, *TEF* promoter-*RPL24* 5′UTR exon–intron-*lacZ*. In all panels, the glc120 medium was used, and each strain was evaluated in biological triplicates (*n* = 3). The bars indicate β-galactosidase activity relative to the control (*pTEF, pTDH1*, and *pTEF* for panels (a), (b), and (c), respectively), with error bars representing the corresponding standard errors. Each bar includes three dots, representing the activity values of the biological triplicates. Statistical analyses were performed using a two-tailed Student’s *t*-test (*P < .05, **P < .01, ***P < .001), with all strains compared to the corresponding no-intron control.

Promoter-proximal introns are known to enhance gene expression [7]. Thus, we inserted introns either immediately downstream of the start codon (location 1) or 11 nucleotides upstream (location 2) within the *lacZ* cassette (Fig. 6a). Compared to the reference no-intron *pTEF* strain, TEFin, I3 (the intron with the highest fluorescence level in our previous test, 139% of the fluorescence of the no-intron *pTEF* strain), and I15 (an intron resulting in 68.7% of the fluorescence of the no-intron *pTEF* strain) at location 1 exhibited β-galactosidase activities of 1.65-fold, 1.92-fold, and 0.81-fold, respectively, consistent with their *hrGFP* expression profiles. Introns inserted at location 2 yielded higher expression: 1.74-fold (TEFin), 2.19-fold (I3), and 1.27-fold (I15), indicating increased expression when introns are positioned within the 5′UTR (Fig. 6a).

To investigate whether intron effects remain synergistic with different promoters, we next used the *pTDH1* cassette (*TDH1* promoter and 5′UTR) to drive *lacZ* expression, inserting introns 15 nucleotides upstream of the start codon, ensuring that the first exon remains sufficiently long to function as an exon [69] and that the upstream “ATT” sequence is preserved [70], consistent with the native intron I9 of *TDH1* (Fig. 6b). Compared to the reference no-intron *pTDH1* strain, insertion of TEFin, I3, and I15 resulted in β-galactosidase activity levels of 1.23-fold, 1.48-fold, and 1.20-fold, respectively (Fig. 6b). Although the activity increase was less pronounced compared to the *pTEF* constructs, the same relative trend in intron effect was observed, indicating consistent intron-mediated regulation with an alternative promoter.

To further test the robustness of intron-mediated regulation, we combined selected introns with different promoters and 5′UTRs by replacing the *TEF* 5′UTR with either the *RPL24* 5′UTR exon (RPLue) alone or the full *RPL24* 5′UTR, which includes both RPLue and the *RPL24* intron (I23). Replacing *TEF* 5′UTR with RPLue alone reduced β-galactosidase activity to 0.84-fold relative to no-intron *pTEF*. However, incorporating the full *RPL24* 5′UTR (RPLue + I23) restored activity to 1.26-fold, highlighting the importance of intron inclusion. Substituting the native I23 with TEFin or I3 resulted in β-galactosidase activity levels of 1.25-fold and 1.54-fold, respectively, confirming intron efficacy within different regulatory sequences and genomic contexts (Fig. 6c).

Collectively, these results highlight that introns directly influence gene expression in *Y. lipolytica*, with the exact effect depending on intron features and location. Moreover, this effect of introns on gene expression proved independent of ORF, genomic location, promoter, and UTR sequence.

## Discussion

Intron-mediated gene regulation is widespread among eukaryotes [7, 71–73], yet the sequence features that determine the regulatory effects of introns remain largely unknown. A better understanding of the intron features that mediate gene expression is not only interesting from a pure biological perspective, but it would also open new avenues toward the use of introns to steer gene expression in various biotechnology applications that rely on genetic elements for tuning gene and protein expression [74–78].

Here, we identified 2421 introns present in 18.1% (1430) of genes in *Y. lipolytica*, including 1302 previously unannotated introns and 479 newly recognized intron-containing genes compared to the latest annotation [6]. Apart from deep sequencing and careful annotation, this substantial increase is explained by examining transcriptomes from 12 diverse conditions, which enabled the discovery of introns in genes that are only expressed in specific conditions or at low levels. The enrichment of introns in housekeeping processes, particularly ribosome biogenesis, has been observed in diverse fungal species [79], including the intron-poor yeast *S. cerevisiae* [14, 17]. In contrast, enrichment in respiration-related pathways appears to be more lineage-specific. In the respiration-dependent yeast *Y. lipolytica*, this enrichment may reflect the essential role of respiratory metabolism in growth. Core intron features reported earlier [6], such as the predominant 5′ splice motif (GTGAGT), 3′ splice motif (CAG/TAG), branch point motif (ACTAAC), and the characteristic 1-nucleotide distance between the branch point and the 3′ splice site, were largely conserved in our expanded dataset. Our dataset also reveals new insights into intron structure and distribution: 5′UTR introns usually contain an upstream ORF (68.3%, 383) and frequently terminate directly upstream of the translational start codon, mirroring the tendency of introns located within the ORF to start immediately downstream of the start codon. Furthermore, 33.6% (481) of intron-containing genes display alternative splicing, a marked increase compared with the 1.8% previously reported [6]. Together, these findings provide a comprehensive view of intron structure and organization across the *Y. lipolytica* genome.

In line with previous studies in other organisms [1, 7], we find that IME is widespread, and in *Y. lipolytica*, intron-containing genes generally show higher and more stable expression than intronless genes. Highly expressed transcripts tend to have introns with high splicing efficiency, and their expression is generally more constitutive across various environments. While it is certainly possible that constitutive housekeeping genes tend to contain introns with higher splicing efficiency, the introns themselves might also contribute directly to the strong constitutive expression, e.g. through coupling of splicing with RNA processing and export [11, 12], as well as through the kinetic buffering effect of multi-step splicing that minimizes transcriptional noise [80]. Our results further demonstrate how intron location and splice site sequences are strongly correlated with splicing efficiency and protein expression, with other intron features, such as distance from the branch point to the 3′ splice site and GC content, also playing a role.

Although we observed that introns can increase both transcript and protein abundance, these readouts reflect the integrated outcome of transcriptional and post-transcriptional regulation. They therefore do not, by themselves, resolve whether IME is driven primarily by increased transcriptional output, altered RNA processing, enhanced mRNA stability, improved nuclear export, increased translational efficiency, or a combination of these mechanisms. Future studies using transcription run-on assays [81] and metabolic RNA-labeling approaches [82] will be important to distinguish the relative contributions of these regulatory layers to IME in *Y. lipolytica*.

Interestingly, intron location is an important factor in shaping gene regulation outcomes [10, 19]. Highly spliced introns with conserved motifs, when inserted immediately downstream of the start codon or in the 5′UTR, markedly increased gene expression. Remarkably, two introns with very low endogenous splicing efficiency (<0.7%) in our assayed conditions still produced significant fluorescence when placed after the start codon, whereas all other introns with similar splicing efficiency showed no expression. As these two introns are the shortest in the set and also maintain the ORF even if they are not spliced out, it is possible that a functional fluorescent reporter may form even without splicing.

Whether splicing is necessary for IME remains unclear [7]. A recent study systematically tested thousands of synthetic introns containing strong splice sites and exhibiting high splicing efficiencies (99.7% with efficiency above 0.9) and found that introns with high splicing efficiency generally conferred strong IME in mammalian cell lines, indicating that efficient splicing is sufficient to drive IME [83]. Here, by extending the range of endogenous intron splicing efficiencies from 0.0001 to 1 while maintaining a constant genomic context, we reveal a strong correlation between endogenous splicing efficiency and gene expression. This suggests that, for most introns, splicing efficiency is similar in native and heterologous contexts, and further strengthens the hypothesis that splicing itself is at the core of IME.

Splicing efficiency correlates with all six examined intron sequence features: the 5′ splice motif, 3′ splice motif, branch point motif, distance from branch point to 3′ splice site, GC content, and intron size. Among these, the three splice-motif features show a clear positive linear correlation with splicing efficiency, indicating that introns with more frequent canonical motifs tend to display higher splicing efficiency. In contrast, the other three features exhibit non-linear correlations: introns with a branch point motif located 0–5 nucleotides upstream of the 3′ splice site display higher splicing efficiency than those with greater distances; introns with a GC content between 0.35 and 0.55 exhibit higher splicing efficiency than those outside this range; and introns 100–300 nucleotides long or longer than 700 nucleotides show lower splicing efficiency than others. Notably, the distribution of splicing efficiency within introns carrying a given optimal sequence feature is consistently bimodal. Analysis of introns exhibiting splicing efficiency below 0.1 shows that 97.6% (706 of 723) contain at least one non-optimal feature, indicating that splicing efficiency is determined by the combined contribution of multiple sequence features. The remaining 2.4% (17 of 723) harbor all six optimal sequence features yet still display low splicing efficiency, suggesting that additional regulatory motifs, such as intronic and exonic splicing enhancers (ISEs/ESEs) or silencers (ISSs/ESSs), may also modulate splicing. These motifs could be systematically investigated through saturation mutagenesis and computational modeling [84, 85]. The characterization of such motifs would further increase our understanding of splicing regulation and facilitate the rational design of synthetic introns for fine-tuning gene expression.

These intron features predict gene expression, with prediction accuracy increasing when constructs are tested in the same genomic environment. Among six sequence features, the 5′ splice motif is the strongest predictor of the effect of introns on expression. The IMEter algorithm, which scores introns by sequence composition, indicates that enhancement-relevant motifs are concentrated toward the 5′ end of introns [86, 87]. We further show that the first six nucleotides of introns (the 5′ splice motif) are the most important sequence feature determining intron- and splicing-dependent effects on gene expression. The 5′ splice motif is recognized through antiparallel complementary base pairing with U1 snRNA, which is encoded by *YALI1_B19143r* and *YALI1_B27339r* in *Y. lipolytica*. Notably, the U1 recognition sequence ACUUAC is highly conserved across diverse eukaryotes [88], whereas predominant 5′ splice motifs vary among species [88–90]. In *Y. lipolytica*, the most abundant motif, GTGAGT (73.0%), is predicted to pair with U1 through Watson–Crick interactions and a single G–U wobble pair [89], while the second most abundant motif, GTAAGT (5.9%), can form complete Watson–Crick pairing. In contrast, GTATGT, the predominant 5′ splice motif reported in other hemiascomycetes [60], accounts for only 2.2% of canonical 5′ splice motifs in *Y. lipolytica*. This motif introduces a U–U mismatch with U1, although such non-canonical interactions can be tolerated during splicing [88, 90]. Together, these observations suggest that distinct yeast lineages have evolved different 5′ splice motif architectures and recognition strategies despite strong conservation of the U1 recognition sequence. In contrast, the most abundant branch point motif (ACTAAC) and the 3′ splice motif (CAG or TAG) in *Y. lipolytica* are relatively conserved among hemiascomycetes [60], supporting the view that changes in the 5′ motif drive much of the lineage-specific variation in splicing.

These global differences in intron architecture provide a framework for interpreting splicing behavior at individual loci in *Y. lipolytica*. We highlight three representative introns from previously investigated *Y. lipolytica* genes, where intron presence or splicing behavior has been noted in gene-focused studies, thereby linking our genome-wide analysis to well-characterized biological contexts. Intron id4686 in *MFE2* contains the predominant splice motif combination GTGAGT–ACTAAC–CAG, consistent with its high mean splicing efficiency across 12 conditions (0.97). This efficient splicing occurs in a biologically relevant locus, as *MFE2* encodes a peroxisomal β-oxidation multifunctional enzyme type 2 with an important role in lipid metabolism in the oleaginous yeast *Y. lipolytica* [67]. In contrast, *EUF1* (*YALI1_F02434g*), which encodes an erythritol utilization factor involved in erythritol catabolism, contains intron id15337 with the splice motif combination GTGAGT–GCTAAC–CAG. Although its branch point motif is suboptimal, this intron is efficiently spliced under endogenous expression conditions, with a mean splicing efficiency of 0.92 and a median abundance of 13.48 TPM. However, previous work showed that strong expression from *pTEF* reduces its splicing efficiency [91], suggesting that splicing can become limiting at high transcript abundance. Finally, *HAC1* (*YALI1_B16808g*), which is involved in the protein-folding stress response, contains a non-canonical intron, id6340, with low mean splicing efficiency (0.20). Consistent with regulated intron removal at this locus, overexpression of *HAC1* alone does not increase the level of the spliced isoform, suggesting that *IRE1*-dependent intron removal is the rate-limiting step [92, 93].

Introns have a very interesting and as yet undervalued potential as regulatory elements that work in concert with more common regulatory elements such as promoters and terminators. Our results help unlock this potential by defining features that determine the regulatory effect of introns. We demonstrate that intron-mediated regulation is tunable, predictable, and robust. Introns can be seamlessly integrated into native gene expression cassettes while preserving existing regulatory elements. This allows their targeted insertion at different genomic loci, with varying splicing efficiencies, to either enhance or reduce gene and protein expression. Moreover, intron pools provide a promising approach to generate mutant strain libraries for functional genomics through random, genome-wide integration—a strategy especially valuable in non-conventional yeasts such as *Y. lipolytica* and *K. marxianus*, where non-homologous end joining is the predominant DNA repair pathway [94, 95]. Taken together, our findings reveal intron features that are highly correlated with intron-mediated gene regulation, paving the way for their application as additional regulatory elements, including the design of novel synthetic introns with specific effects on gene expression.

## Supplementary Material

gkag650_Supplemental_Files

## Data Availability

All data supporting the findings of this study are available within the main article and its Supplementary data. Source data are provided with this paper and/or deposited in Zenodo at https://doi.org/10.5281/zenodo.17464154. RNA sequencing data have been deposited in the NCBI Sequence Read Archive (SRA) under accession code PRJNA1346563. The custom code used for putative intron identification and filtration, GO enrichment analysis, branch point motif identification, consensus score calculation of splicing motifs, and linear regression model development has also been deposited in Zenodo at https://doi.org/10.5281/zenodo.17464154.
