# Supplementary material for: Intron location and sequence modulate gene expression in Yarrowia lipolytica
Source: Nucleic Acids Res. 2026 Jun 27;54(12):gkag650. doi: 10.1093/nar/gkag650 (PMC13309789; doi:10.1093/nar/gkag650)
Supplement: gkag650_Supplemental_Files [file gkag650_supplemental_files.zip › Supplementary Figures.pdf]

Supplementary Figures for

Intron location and sequence modulate gene expression in *Yarrowia lipolytica*

AUTHORS

Qi Qi[1,2], Pedro Tomaz da Silva[3,4], Vasileios Vangalis[1,2], Seppe Dockx[1,2], Jan Steensels[1,2], Karin Voordeckers[1,2], Julien Gagneur[3,5,6,*], Kevin J. Verstrepen[1,2,*]

[1] Centre of Microbial and Plant Genetics (CMPG), Department of Microbial and Molecular Systems (M^{2}S), KU Leuven, Leuven, 3000, Belgium.

[2] Lab for Systems Biology, VIB Center for Microbiology, VIB, Leuven, 3001, Belgium.

[3] School of Computation, Information and Technology, Technical University of Munich, Munich, 80333, Germany.

[4] Munich Center for Machine Learning, Munich, 80333, Germany.

[5] Institute of Human Genetics, School of Medicine, Technical University of Munich, Munich, 81675, Germany.

[6] Computational Health Center, Helmholtz Center Munich, Neuherberg, 85764, Germany.

* To whom correspondence should be addressed. Email: Kevin.Verstrepen@kuleuven.be

Correspondence may also be addressed to Julien Gagneur. Email: Gagneur@in.tum.de

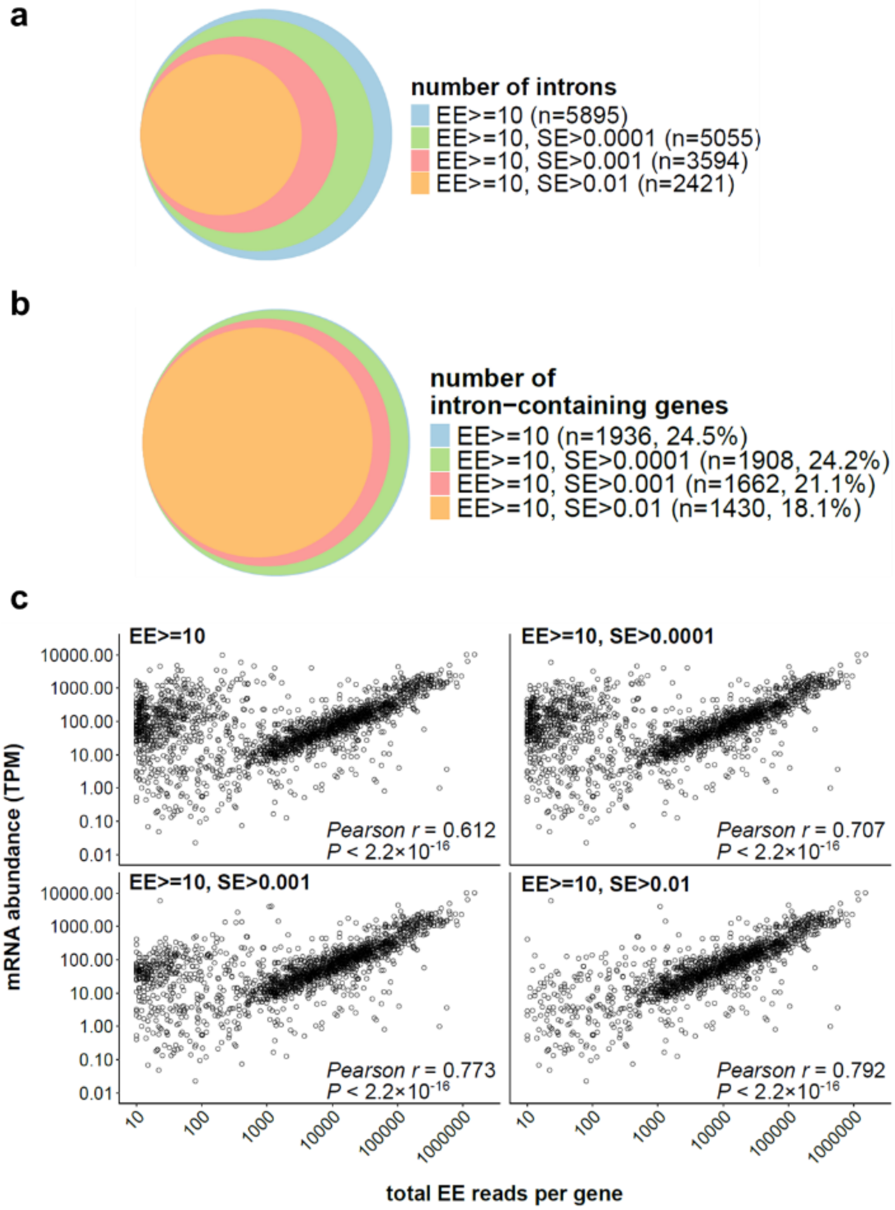

Fig. S1. Both exon junction reads (EE) and splicing efficiency (SE) were used as thresholds for intron filtering. Both EE and SE were computed using pooled data across all samples. **a**) Number of introns retained under four different filtering thresholds. The **n** after each threshold indicates the number of introns retained when applying that threshold. **b**) Number of intron-containing genes retained under four filtering thresholds. For each threshold, **n** indicates the number of intron-containing genes, and the percentage shows its proportion of all genes in the genome. **c**) The most stringent threshold, ‘EE>=10, SE>0.01’, was used as the primary threshold in this study. Scatter plots show the Pearson correlation between total EE reads per gene and corresponding mRNA abundance under four different intron filtering thresholds. mRNA abundance is represented as transcripts per million (TPM), using median TPM across 12 conditions.

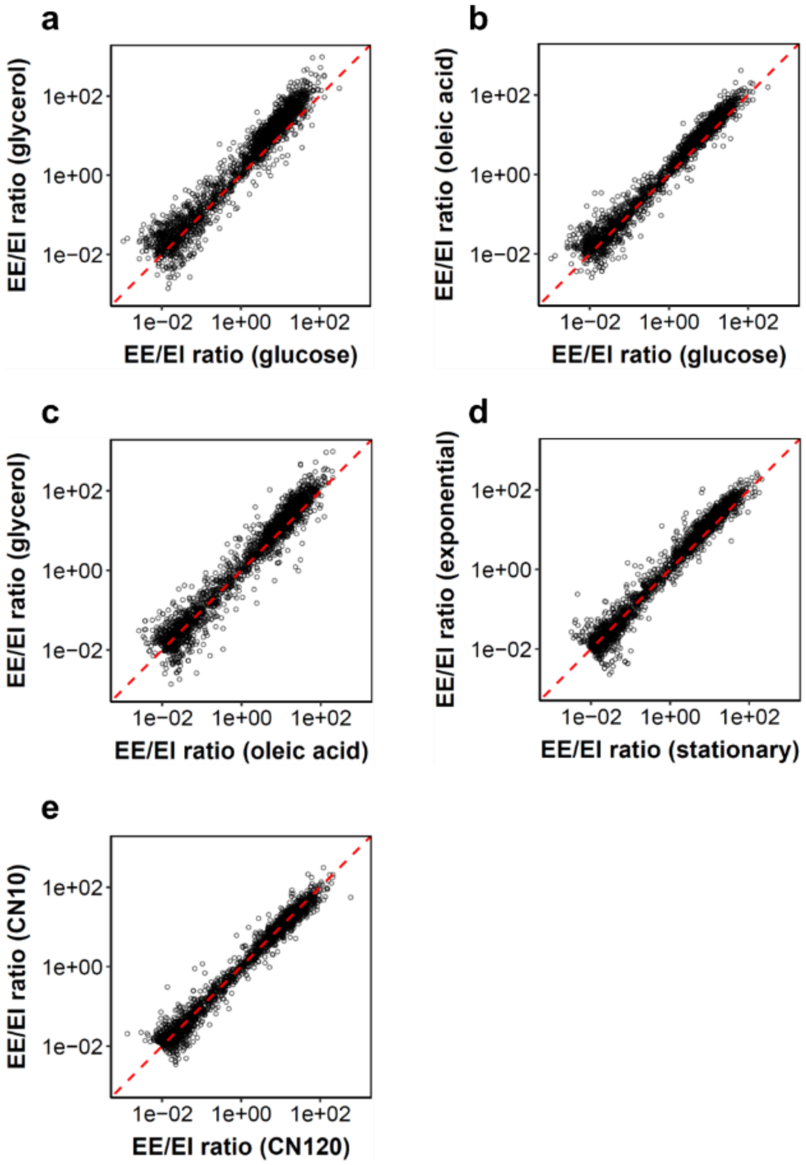

Fig. S2. Splicing efficiency variation across conditions is broadly consistent among introns. In all panels, both EE and EI values represent sums obtained from pooled data across conditions relevant to the indicated carbon source, growth phase, or C/N ratio.

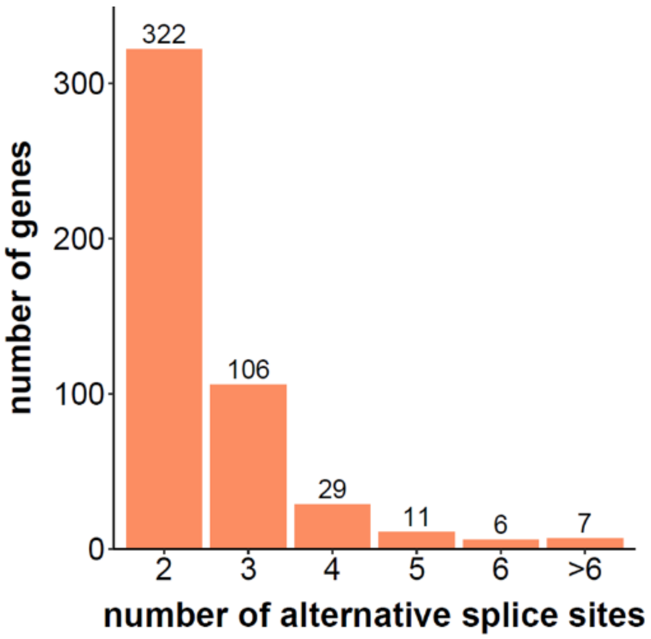

Fig. S3. 481 intron-containing genes show alternative splice sites.

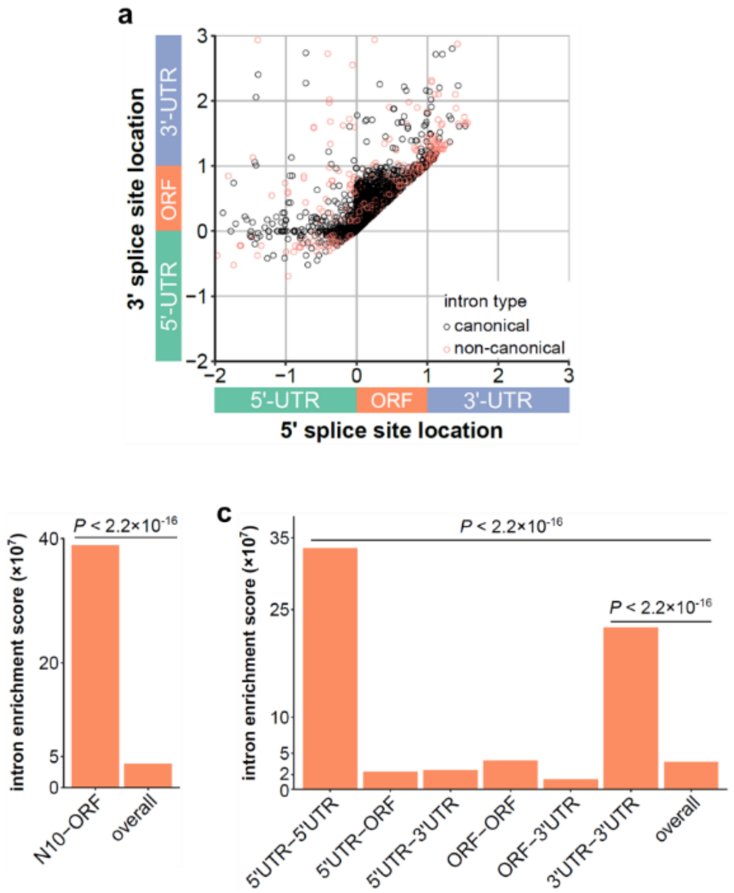

Fig. S4. Introns are enriched at the ORF start and throughout the UTRs. a) Overview of intron location relative to the gene. Gene ORF size was normalized to 1. Canonical introns are defined as those with GT-AG dinucleotides at the 5′ and 3′ splice sites. All others are considered non-canonical introns. All 2,421 introns are located within 2× the length of the gene's ORF upstream of the start codon or downstream of the stop codon. Introns can be categorized into six types based on the relative locations of their 5′ and 3′ splice sites: 5′UTR-5′UTR, 287 introns; 5′UTR-ORF, 250 introns; 5′UTR-3′UTR, 24 introns; ORF-ORF, 1640 introns; ORF-3′UTR, 100 introns; 3′UTR-3′UTR, 120 introns. Red circles represent non-canonical introns, black circles represent canonical introns. **b**) Introns are enriched within the first 10 nucleotides of the ORF compared to the overall intron distribution. N10-ORF means introns with 5′ splice site (5′ss) in the first 10 nucleotides of the ORF, and 3′ splice site (3′ss) in the ORF. The 'overall' category includes all introns. **c**) Introns are enriched within the full 5′UTR, and the full 3′UTR. Intron types were classified as follows: 5′UTR-5′UTR (both splice sites in the 5′UTR), 5′UTR-ORF (5′ss in the 5′UTR, 3′ss in the ORF), 5′UTR-3′UTR (5′ss in the 5′UTR, 3′ss in the 3′UTR), ORF-ORF (both splice sites in the ORF), ORF-3′UTR (5′ss in the ORF, 3′ss in the 3′UTR), and 3′UTR-3′UTR (both splice sites in the 3′UTR). UTRs were restricted to the 200 bp regions upstream or downstream of the ORF. In panels **b** and **c**, the intron enrichment score was calculated as the ratio of the observed number of introns of each type - defined by the relative positions of their 5′ and 3′ splice sites - to the total number of possible introns of that type. Statistical significance was performed using the Chi-square test.

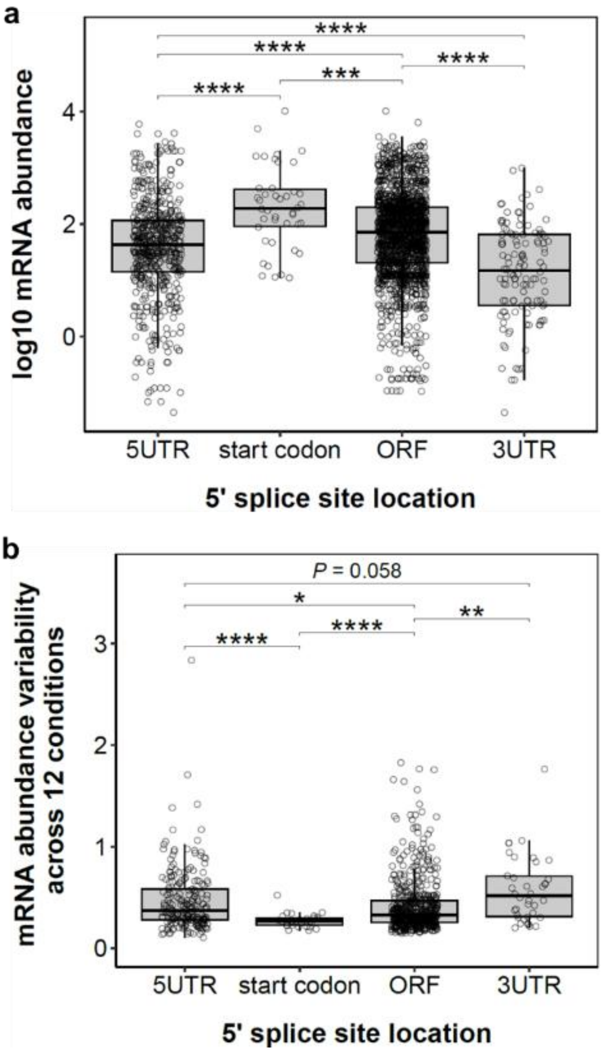

Fig. S5. Gene expression level and variability vary with intron location. Genes containing start codon introns tend to have **a**) highest splicing efficiency and **b**) lowest expression variability across 12 conditions than others. In both panels, mRNA abundance is represented as TPM using median TPM across 12 conditions. Statistical analysis was performed using a two-tailed Welch's t-test (unequal variance) (* $P < 0.05$, ** $P < 0.01$, *** $P < 0.001$, **** $P < 0.0001$).

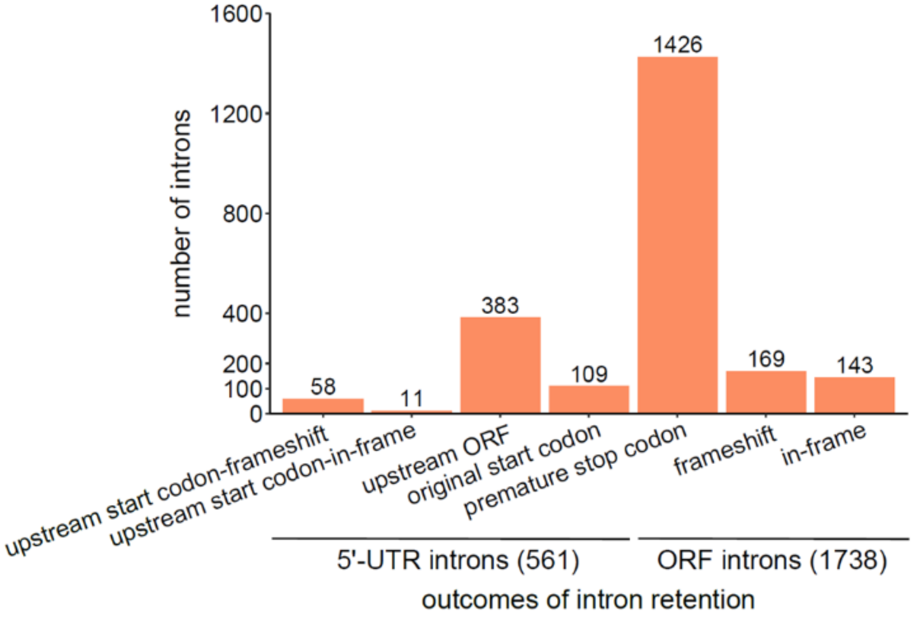

Fig. S6. 75% of intron retention (1,809 out of 2,421) would lead to upstream ORF or premature stop codon. The presence of upstream start codons and stop codons in 5′-UTR introns or premature stop codons in ORF introns were examined. Classification: upstream start codon-frameshift, the intron contains an upstream start codon and would cause a frameshift mutation if translation starts here; upstream start codon-in-frame, an in-frame upstream start codon; upstream ORF, the intron contains both an upstream start codon and a stop codon, potentially forming an upstream ORF; original start codon, the original start codon is the first start codon found; premature stop codon, a premature stop codon; frameshift, the intron does not contain a stop codon and would cause a frameshift mutation; in-frame, the intron is in-frame and does not introduce a stop codon.

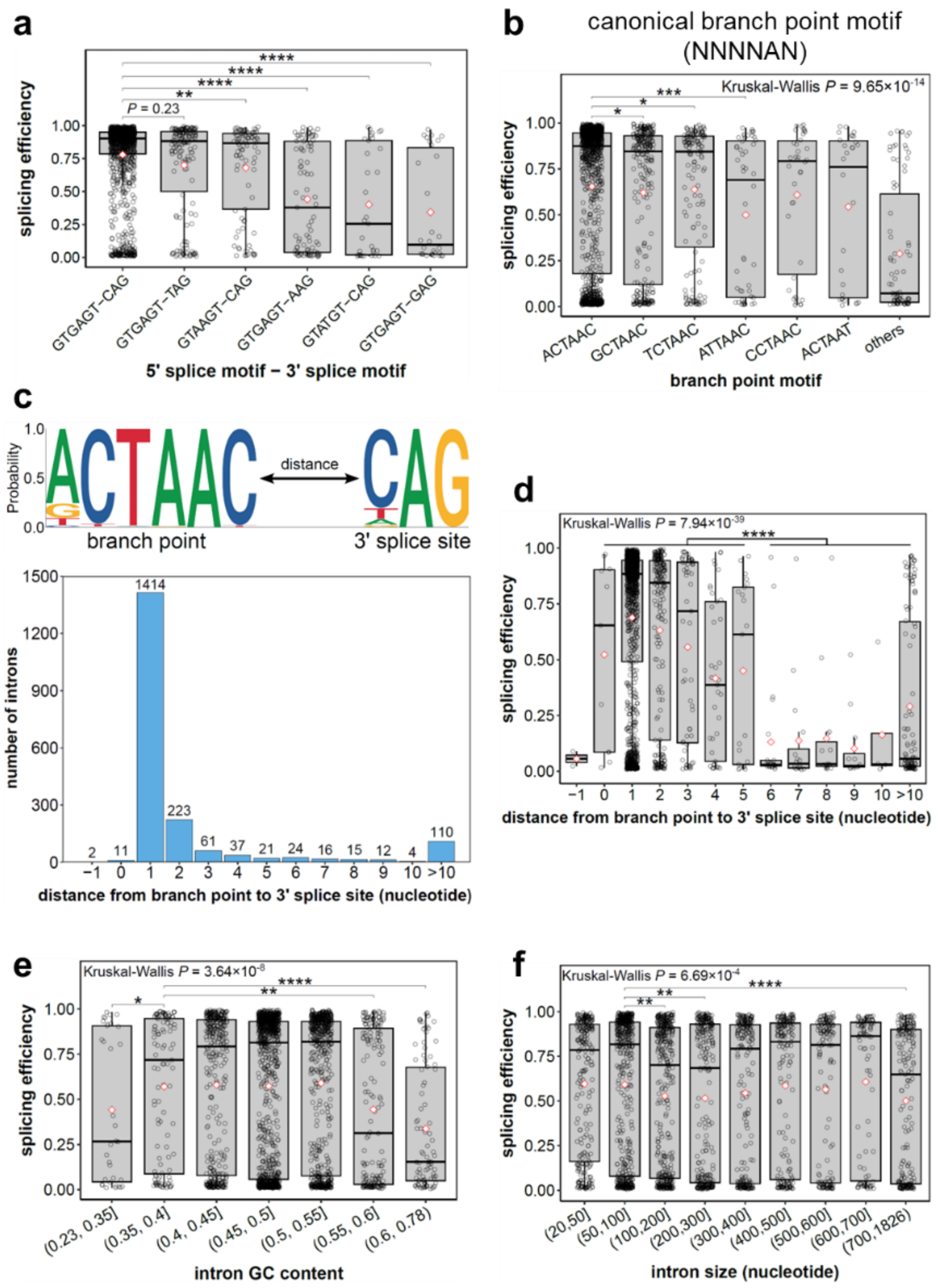

Fig. S7. Splicing efficiency is correlated with intron sequence features. Splice site combination, branch point, distance from branch point to 3′ splice site, intron GC content, and intron size are examined. **a**) Introns with the GTGAGT–CAG or GTGAGT–TAG splice site combination tend to exhibit the highest splicing efficiency. Box plots display the splicing efficiency distributions for the six most common canonical 5′–3′ splice site motif combinations. Intron splicing efficiency is represented as the mean splicing efficiency across all conditions. Intron counts per box (left to right): 1185, 162, 89, 91, 33, 30. **b**) Branch point motif correlates with splicing efficiency. Intron splicing efficiency is represented as the mean splicing efficiency across all conditions. The protocol used for identification of potential branch point motifs is shown in Methods section. Intron counts per box (left to right): 1317, 286, 147, 50, 38,

30, 82. **c**) 70% of canonical introns have a branch point-to-3′ splice site distance of only 1 nucleotide. The sequence logo illustrates the branch point and 3′ splice site motif composition across all canonical introns identified, alongside the measured distances shown in the accompanying bar plot. A distance of -1 indicates that both motifs share a nucleotide, which is a cytosine. **d**) Branch point-to-3′ splice site distance correlates with splicing efficiency. Intron splicing efficiency is represented as the mean splicing efficiency across all conditions. Intron counts per box are shown in panel c. **e**) Intron GC content correlates with splicing efficiency. A 0.05 sliding window was applied, with adjacent groups merged if each contained fewer than 30 introns. Intron splicing efficiency is represented as the mean splicing efficiency across all conditions. Intron counts per box (left to right): 34, 111, 372, 1013, 617, 180, 94. **f**) Intron size correlates with splicing efficiency. Intron splicing efficiency is represented as the mean splicing efficiency across all conditions. Intron counts per box (left to right): 259, 553, 419, 285, 234, 193, 156, 105, 217. In panels **a**, **b**, and **d**-**f**, intron splicing efficiency is represented as the mean splicing efficiency across all conditions. For each intron subset, the mean splicing efficiency is indicated by a red diamond. Statistical analyses between subsets were performed using the Wilcoxon rank-sum test (* $P < 0.05$, ** $P < 0.01$, *** $P < 0.001$, **** $P < 0.0001$), and overall group differences were assessed using the Kruskal-Wallis test.

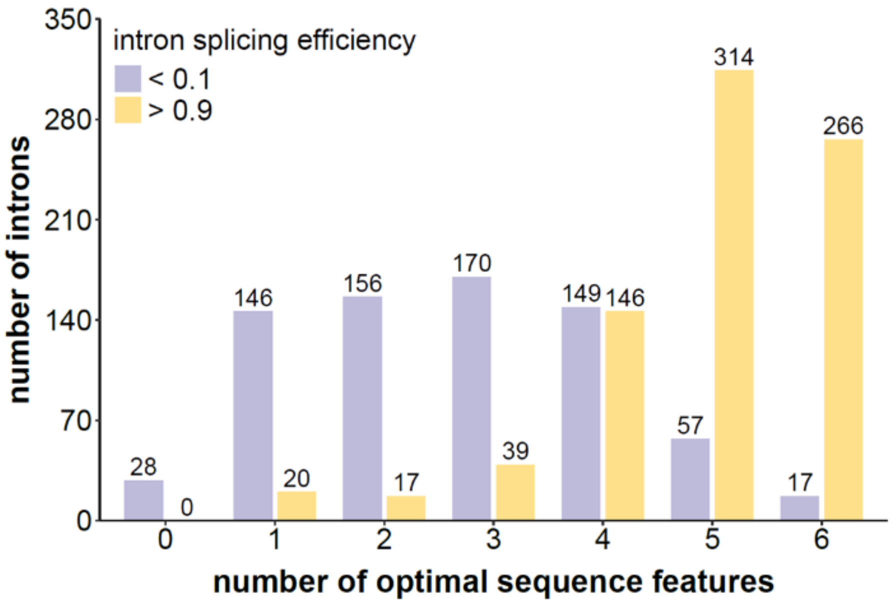

Fig. S8. Intron splicing efficiency correlates with the number of optimal sequence features that the intron contains. All six examined intron sequence features are shown, including 5′ splice motif, 3′ splice motif, branch point motif, distance from branch point to 3′ splice site, intron GC content, and intron size. Intron splicing efficiency is represented as the mean splicing efficiency across all conditions.

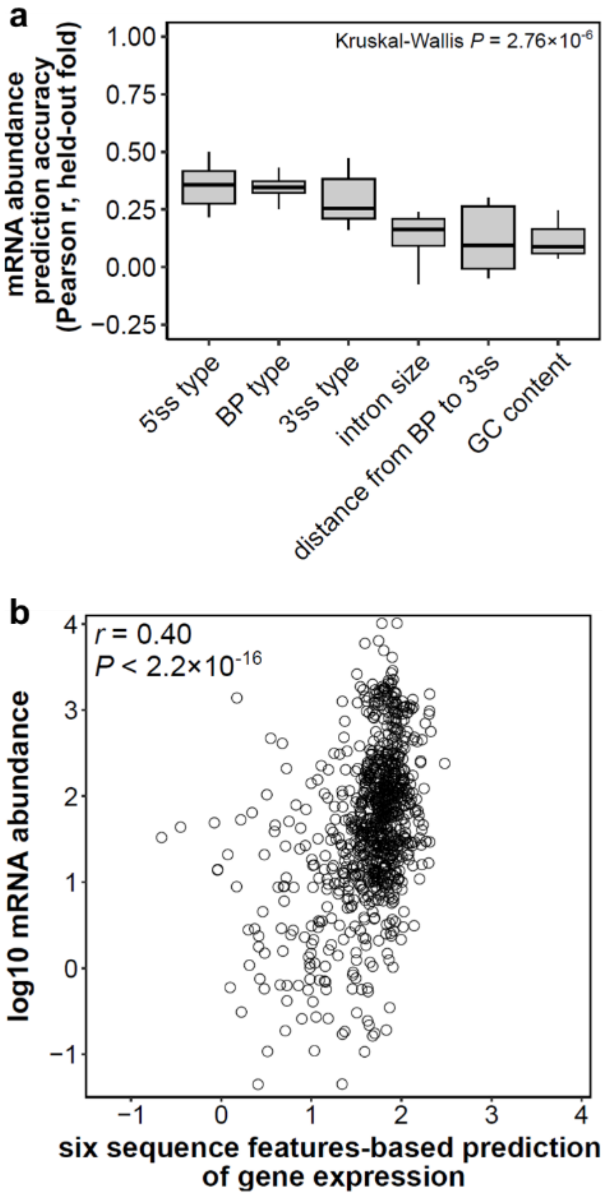

Fig. S9. The six intron sequence features predict native intron-containing gene expression *in situ*. a) All six features show varying degrees of positive correlation with gene expression. Box plots display the predictive performance of six individual intron features, each used in a separate linear regression model to predict mRNA abundance, evaluated across ten-fold cross-validation. 864 single-intron genes were included. Statistical analysis was performed using the Kruskal-Wallis test. **b**) The six sequence features-based linear regression model was applied to predict *in situ* expression of 864 single-intron genes and yielded a Pearson correlation of 0.40. Evaluation was performed across ten cross-validation folds. In both panels, mRNA abundance is represented as the median TPM across 12 conditions.

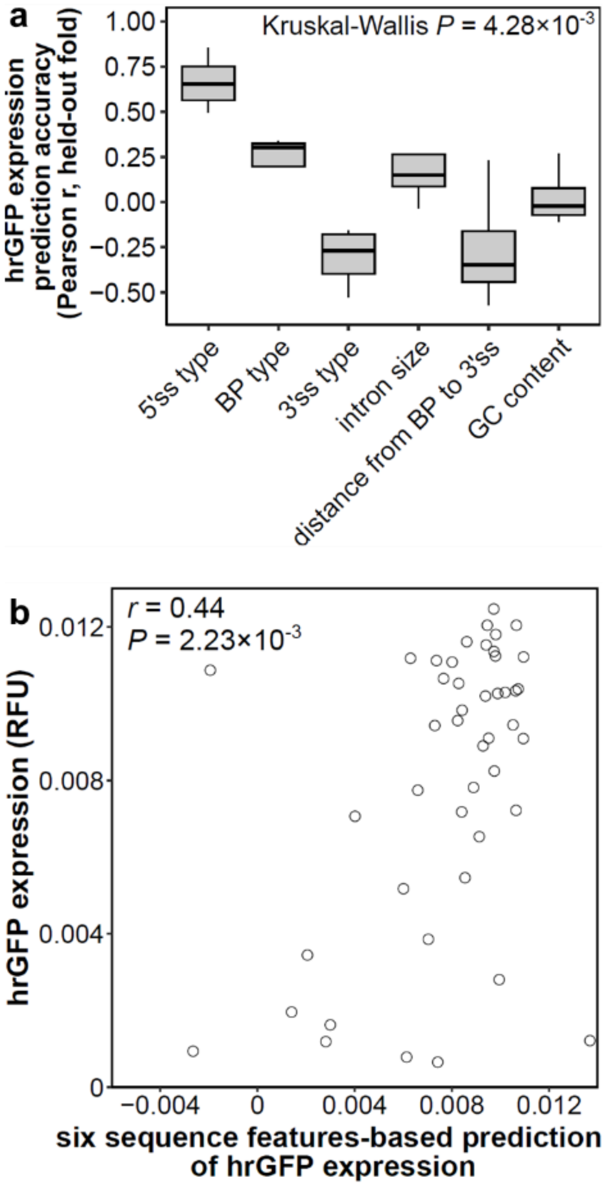

Fig. S10. The six intron sequence features predict *hrGFP* expression. a) All six features show varying degrees of correlation with *hrGFP* expression. Box plots display the predictive performance of six individual intron features in separate linear regression models for *hrGFP* expression, evaluated across four-fold cross-validation. Each model was trained using a single feature. 46 strains containing selected introns from the 'EE>=10, SE>0.01' group or the *TEF* intron are included. Statistical analysis was performed using the Kruskal-Wallis test. **b**) The six sequence features predict *hrGFP* expression with a Pearson correlation of 0.44. Evaluation was performed across four cross-validation folds.
